# Metabolic Engineering Strategies in Diatoms Reveal Unique Phenotypes and Genetic Configurations With Implications for Algal Genetics and Synthetic Biology

**DOI:** 10.3389/fbioe.2020.00513

**Published:** 2020-06-05

**Authors:** Jestin George, Tim Kahlke, Raffaela M. Abbriano, Unnikrishnan Kuzhiumparambil, Peter J. Ralph, Michele Fabris

**Affiliations:** ^1^University of Technology Sydney, Climate Change Cluster, Faculty of Science, Ultimo, NSW, Australia; ^2^CSIRO Synthetic Biology Future Science Platform, Brisbane, QLD, Australia

**Keywords:** microalgae, *Phaeodactylum tricornutum*, extrachromosomal expression, random integration, long-read sequencing, integration islands, heterologous monoterpenoids, synthetic biology

## Abstract

Diatoms are photosynthetic microeukaryotes that dominate phytoplankton populations and have increasing applicability in biotechnology. Uncovering their complex biology and elevating strains to commercial standards depends heavily on robust genetic engineering tools. However, engineering microalgal genomes predominantly relies on random integration of transgenes into nuclear DNA, often resulting in detrimental “position-effects” such as transgene silencing, integration into transcriptionally-inactive regions, and endogenous sequence disruption. With the recent development of extrachromosomal transgene expression via independent episomes, it is timely to investigate both strategies at the phenotypic and genomic level. Here, we engineered the model diatom *Phaeodactylum tricornutum* to produce the high-value heterologous monoterpenoid geraniol, which, besides applications as fragrance and insect repellent, is a key intermediate of high-value pharmaceuticals. Using high-throughput phenotyping we confirmed the suitability of episomes for synthetic biology applications and identified superior geraniol-yielding strains following random integration. We used third generation long-read sequencing technology to generate a complete analysis of all transgene integration events including their genomic locations and arrangements associated with high-performing strains at a genome-wide scale with subchromosomal detail, never before reported in any microalga. This revealed very large, highly concatenated insertion islands, offering profound implications on diatom functional genetics and next generation genome editing technologies, and is key for developing more precise genome engineering approaches in diatoms, including possible genomic safe harbour locations to support high transgene expression for targeted integration approaches. Furthermore, we have demonstrated that exogenous DNA is not integrated inadvertently into the nuclear genome of extrachromosomal-expression clones, an important characterisation of this novel engineering approach that paves the road to synthetic biology applications.

## Introduction

Diatoms are a diverse group of unicellular Stramenopile microalgae that have received substantial attention for their ecological importance (Armbrust, 2009) and biotechnological potential (Huang and Daboussi, 2017). Newly developed genetic resources hold much promise for diatom functional genetics studies and have propelled the model pennate diatom *Phaeodactylum tricornutum* into the field of synthetic biology. Firstly, next-generation genetic engineering tools have now been established in *P. tricornutum*. This includes targeted genetic engineering via transcription activator-like effector nucleases (TALENs) (Daboussi et al., 2014; Weyman et al., 2015; Serif et al., 2017) and CRISPR-Cas9 (Nymark et al., 2016; Serif et al., 2018; Sharma et al., 2018) techniques with wide applications for biotechnology and basic research. Next-generation engineering strategies also include the recently demonstrated extrachromosomal approach. Here, potentially large episomes that contain various DNA parts can be maintained and expressed without requiring genomic integration (Karas et al., 2015a). Consequently, extrachromosomal transformation is anticipated to become increasingly widely-used in diatom genetic engineering (Huang and Daboussi, 2017). These next-generation engineering strategies are central to diatom genetics and synthetic biology primarily because they allow multi-gene stacking approaches (Goyal et al., 2009; Ainley et al., 2013). Secondly, the recently developed Universal Loop (uLoop) assembly kit provides a collection of useful parts for modular DNA assembly and high-throughput testing as well as more complex gene-stacking designs for *P. tricornutum* and other diatoms (Pollak et al., 2019). Altogether, these resources offer unparalleled potential of diatoms such as *P. tricornutum* compared to other model algal chassis.

Further to these developments, intrinsic desirable biological traits have elevated this microbe as a promising alternative to well-established chassis, *Escherichia coli* and *Saccharomyces cerevisiae*. Such traits include its robustness and scalability for industrial-scale growth (Hamilton et al., 2015); and—unlike bacteria and yeast species—its ability to fix carbon via photosynthesis for cheaper culture conditions. There is also an increased availability of transcriptomic, metabolomic, and proteomic datasets for uncovering previously unknown traits in diatoms (Ashworth et al., 2016; Longworth et al., 2016; Remmers et al., 2018; Smith et al., 2019) including unique aspects with potential biotechnological relevance (Kroth et al., 2008; Allen et al., 2011; Fabris et al., 2012, 2014).

*Phaeodactylum tricornutum* is poised to become a widely-used, reliable chassis organism and has been validated in various high interest biotechnological applications, including in the production of bioplastic precursor compounds (Hempel et al., 2011a), therapeutic antibodies (Hempel et al., 2011b; Hempel and Maier, 2012), biofuels (Yao et al., 2014) and nutritional supplements (Hamilton et al., 2014). However, these approaches have all relied on the first generation genetic engineering strategy of randomly integrated chromosomal expression (RICE) of exogenous DNA. While RICE has been crucial for generating a myriad of transgenic *P. tricornutum* strains, both for basic (Lavaud et al., 2012; Liu et al., 2016) and applied research (Hempel and Maier, 2012); it is beset with silencing issues resulting in low transgene expression and stability (Cerutti et al., 2011). This is largely attributed to the “position effect” phenomenon, whereby transgenes integrate into regions in the genome that are unfavourable for transgene expression (Gangl et al., 2015; Doron et al., 2016; Huang and Daboussi, 2017), such as transcriptionally repressed regions (Elgin, 1996). In microalgal research, there is virtually no information regarding the mechanisms driving and regulating RICE. Uncovering this knowledge is important for better understanding of a strategy that is still widely used today, including for CRISPR-Cas9 targeted genome editing (Hopes et al., 2016; Nymark et al., 2016; Greiner et al., 2017).

In order for next-generation engineering tools to deliver a variety of synthetic biology applications in *P. tricornutum* and to replace RICE, they need to be better characterised. For example, even though targeted integration has been demonstrated in this species (Weyman et al., 2015), there are no known safe harbour loci–regions in the nuclear genome that facilitate stable, high transgene expression–identified in *P. tricornutum* or any other eukaryotic photosynthetic microbe. Similarly, non-integrative episomes are extremely appealing for synthetic biology applications as backbones of self-maintaining mini-chromosomes. However, there is little knowledge available regarding mechanisms of episomal maintenance (Diner et al., 2016), including the level of transgene expression that can be achieved by extrachromosomal expression (EE), and whether fragments of episomal DNA are inadvertently integrated into the nuclear genome. This is because EE technology has only recently been described in diatoms with limited examples of its use to express transgenes. Resolving these knowledge gaps will enable understanding of how different genetic engineering strategies may alter the diatom's biology and DNA integration patterns for developing more reliable next-generation engineering approaches required for complex synthetic biology.

Given the developments for diatom synthetic biology, *P. tricornutum* is now being explored for its potential for heterologous terpenoid production (Vavitsas et al., 2018). *Phaeodactylum tricornutum* is a promising alternative chassis compared to the more widely used bacteria and yeast species, which require extensive engineering to increase relatively low flux to isoprenoid biosynthesis (Zurbriggen et al., 2012; Paddon et al., 2013; Bian et al., 2017; Wang et al., 2017). Until recently, *P. tricornutum* had only been metabolically engineered to produce heterologous terpenoids betulin and lupeol (D'Adamo et al., 2018) by RICE. However, we have since demonstrated the first use of EE for metabolic engineering in *P. tricornutum* (Fabris et al., 2020) by expressing a geraniol synthase from the medicinal plant *Catharanthus roseus* (EC 3.1.7.11, *CrGES*) for the heterologous production of geraniol. Geraniol is a commercially relevant monoterpenoid with a variety of applications as flavourant, fragrances, and insect repellent (Chen and Viljoen, 2010). Geraniol is also the first intermediate in the monoterpenoid indole alkaloids (MIAs) biosynthesis pathway, which in *C. roseus* leads to the synthesis of the very high-value products with pharmaceutical applications (Van Moerkercke et al., 2013; Caputi et al., 2018).

Herein, with the aim of laying the basis for more sophisticated synthetic biology and metabolic engineering strategies, we generated and thoroughly profiled libraries of transgenic *P. tricornutum* cell lines engineered to produce geraniol via either EE or RICE. By adopting high-throughput phenotyping, we unveiled important intrinsic differences both between and within EE and RICE cell lines. This revealed a small selection of high-performing RICE strains, as well as a highly consistent transgene expression phenotype across EE strains. We used long-read DNA sequencing to interrogate the genomes of selected EE and RICE lines. Our results provide a complete analysis of all integration events, genomic locations, and transgene arrangements associated with high-performing RICE strains at both genome-wide and subchromosomal scale, never reported before in any microalga, and confirmed the non-integrative nature of EE.

Our findings are key for understanding the underexplored dynamics of EE in diatoms, to be used as the basis for gene-stacking based synthetic biology applications such as metabolic pathways and complex genetic circuit assembly and expression. They also highlight the importance of moving to next-generation genetic engineering strategies in *P. tricornutum* and lay the groundwork for identifying putative genomic safe harbour locations that support high transgene expression for targeted integration strategies.

## Methods

### Microbial Strains and Growth Conditions

*Phaeodactylum tricornutum* CCAP1055/1 was grown in liquid ESAW (Berges et al., 2001) supplemented with 50 μg/mL zeocin (Invivogen, San Diego, CA, USA) where appropriate, under 100 μE m^−2^ s^−1^ light in 21 °C shaking at 95 rpm. *Phaeodactylum tricornutum* induction media was prepared following ESAW protocol but without any addition of phosphate. *Escherichia coli* was grown in Luria broth supplemented with 100 μg/mL ampicillin.

### Cloning and Genetic Construct Assembly

Plasmids and episomes were constructed using Gibson assembly cloning kit (New England Biolabs, Hitchin, UK). Plasmids were propagated in *E. coli* strain Top10 and purified by Monarch Plasmid Miniprep Kit (New England Biolabs, Hitchin, UK). PCR amplification was performed using Q5 high fidelity polymerase (New England Biolabs, Hitchin, UK) and PCR screening was performed using GoTaq Flexi DNA polymerase (Promega, Wisconsin, United States) according to the manufacturer's instructions. Plasmid coding sequences were validated by Sanger sequencing (Macrogen Korea, Seoul, Korea). Episomes *pPtPBR11_AP1p_CrGES-mVenus* (mVenus NCBI Accesssion: AAZ65844.1) and a control *pPtPBR11_AP1p_mVenus* are described in Fabris et al. (2020). Expression plasmid for chromosomal integration, *pUC19_AP1p_CrGES-mVenus*, was ligated by Gibson assembly into the pUC19 cloning vector linearised with BamHI. Both the *AP1p_CrGES-mVenus_FCBPt* expression cassette and the *FCBPp_ShBle_FCBPt* zeocin resistant cassette were amplified from *pPtPBR11_AP1p_CrGES-mVenus* using 5′-tcgagctcggtacccgggCTAACAGGATTAGTGCAATTC-3′ forward primer and 5′-aggtcgactAGACGAGCTAGTGTTATTC-3′ reverse primer; and 5′-agctcgtctAGTCGACCTGCACATATG-3′ forward primer and 5′-tgcaggtcgactctagagAGACGAGCTAGTGTTATTC-3′ reverse primer, respectively. Similarly, *pUC19_AP1p_mVenus* expression plasmid for genomic integration was ligated by Gibson assembly into the pUC19 cloning vector linearised with BamHI. The *AP1p_mVenus_FCBPt* expression cassette was amplified using the same primers for amplifying *AP1p_CrGES-mVenus_FCBPt* expression cassette from *pPtPBR11_AP1p_mVenus* episome. Sequences of the vectors used in this work are provided in Supplementary File 2.

### Diatom Transformation and Conjugation

*Phaeodactylum tricornutum* was transformed by biolistic bombardment using PDS-1000/He System with Hepta adapter (Bio-Rad) for nuclear genomic integration of plasmid DNA (Kroth, 2007). Afterward, the cell mixture was left to recover 12 h before being scraped and plated onto fresh ½ ESAW 50 μg/mL zeocin agar plates and left for 4–5 weeks for single colonies to appear. *E. coli* containing *pTA-Mob* (Karas et al., 2015a) and *pPtPBR11_AP1p_CrGES-mVenus* or *pPtPBR11_AP1p_mVenus* plasmids (Fabris et al., 2020) were used for conjugation with *P. tricornutum* according to protocol described by Diner et al. (2016). The cell mixture was scraped and plated onto 3–5 fresh ½ ESAW zeocin agar plates and left for 10–15 days when single colonies appeared. Single colonies generated by biolistic bombardment and conjugation were picked and inoculated into individual wells of 96-well round bottom plates containing 200 μl of ESAW supplemented with 50 μg/mL zeocin. The EE and RICE generated cell lines were incubated at 21°C with 100 μE m^−2^ s^−1^ light for 1 week to adjust to liquid growth, after which they were subcultured every 4 days. For high-throughput screening, cell lines were subcultured over 3 weeks in ESAW supplemented with 50 μg/mL zeocin (or without supplementation for stability analysis), and induced with phosphate-free ESAW for 24 h before flow cytometry.

### Flow Cytometry and Fluorescence-Activated Cell Sorting (FACS)

Once antibiotic resistant single colonies were established in liquid culture, they were used to inoculate fresh 200 μL of ESAW with and without zeocin. Cell lines were subcultured 1:7 (v:v) every 4 days for 3 weeks under these conditions. On day 4 of culture, plates were centrifuged at 2,300 g for 3 min to pellet cells. The supernatant was removed and cells were washed with 150 μL induction media twice before being resuspended in 200 μL of induction media and induced for 24 hrs. Induced cells were screened by flow cytometry using CytoFLEX S (Beckman Coulter). Fluorescence was excited using a 488 nm laser. mVenus fluorescence was detected using a 525/40 nm filter and chlorophyll fluorescence was detected using 690/50 nm filter. Compensation of chlorophyll channel was set to 0.3. The distribution of eight single colonies of wild type *P. tricornutum* cultured and induced in the same way as transgenic cell lines were used as controls to determine the mVenus auto fluorescence for screening. Only chorophyll positive cells were included in the analysis to account for cell debris and other background events.

The per cell mVenus fluorescence of 20,000 events was log normalised and violin plots were created in Python using the seaborn data visualisation library (v. 0.9.0) (Allen et al., 2018). Cell lines selected for geraniol production analysis were sorted using BD Influx FACS (BD Biosciences). All cell lines were cultured and induced as described above prior to FACS. Yellow mVenus fluorescence was detected using a 488 nm laser for excitation and a 530/40 nm filter. Wild type *P. tricornutum* cultured and induced in the same way as the transformants, was used as a control to determine the mVenus auto fluorescence distribution. A preliminary screen of a pooled sample of the top eight RICE_mV transformants was used to define the mVenus positive gate which did not overlap with the wild type control. One thousand cells from each transformant cell line that fell into this gate were collected into 96-well round bottom plate wells containing 200 μL ESAW media. After sorting, cells were incubated for 1–2 weeks in 200 μL ESAW supplemented with zeocin 50 μg/mL in 96-well round bottom plate, after which they were subcultured as described previously.

### Geraniol Capture and Analysis

Cell lines analysed for geraniol production were scaled up to 50 mL preculture in ESAW supplemented with zeocin 50 μg/mL. Precultures were used to inoculate 50 mL fresh ESAW media without zeocin in 250 mL shake flasks at 10,000 cells/mL density on Day 0. Cultures in late-exponential phase were induced in the presence of isopropyl myristate (C_17_H_34_O_2_) to capture volatile monoterpenoids (Jiang et al., 2017). On Day 4 the total culture was collected and centrifuged at 3,000 g for 4 min to pellet cells. Cells were resuspended in 4 mL induction media and washed twice in induction media before being resuspended in 30 mL induction media in fresh 250 mL shake flasks with 1.6 mL isopropyl myristate, which was harvested 72 h after induction and stored −80°C until being analysed by GCMS. Geraniol was captured, sampled and analysed as described in Fabris et al. (2020).

### High Molecular Weight Genomic DNA (gDNA) Extraction

Genomic DNA from *P. tricornutum* transformants was extracted using 7 × 10^7^ cells in 4–6 extractions to obtain ~7.5 μg high molecular weight, purified gDNA (dx.doi.org/10.17504/protocols.io.qzudx6w). The DNA was resuspended in 25–45 μL ultrapure water overnight at room temperature.

### MinION Sequencing

MinION sequencing libraries were prepared according to the 1D Genomic DNA by ligation (SQK-LSK108) protocol supplied by the MinION manufacturer (Oxford Nanopore Technologies) with modifications. Briefly, the DNA fragmentation step was replaced with two bead-cleaning steps. An initial 1:0.1 bead clean of gDNA to GC Biotech CleanNGS (CNGS-0005) beads was performed and the sample was gently mixed by flicking, and was slowly repeatedly inverted for 5 min, and pelleted on a magnetic rack to collect the supernatant. A second 1:1 bead clean was performed using the supernatant and beads. The sample was gently mixed by flicking, incubated by slowly rotating by hand for 5 min, and pelleted on a magnetic rack. The supernatant was removed and DNA bound to the beads was washed with 200 μL freshly prepared 70% ethanol twice without removing the tube off the magnet or disturbing the pellet. The beads and DNA were resuspended in 46 μL ultrapure water, incubated at room temperature for 5 min, the beads were pelleted on the magnet and the supernatant containing the DNA was collected and used according to the manufacturer protocol. Samples were sequenced until a coverage of seven to ten times was achieved. Raw reads were base called and quality filtered using albacore v2.2.6 with default settings. All the sequencing data has been deposited in NCBI BioProject under the ID PRJNA593624.

### Identifying Transgene Integration Locations in Nuclear Genome

To identify reads which contain RICE plasmid DNA and remove reads made up of genomic DNA only, all reads were aligned against *pUC19_AP1p_CrGES-mVenus* RICE plasmid using BLAST. Reads that did align to RICE plasmid were defined as initial hits. To account for regions in *pUC19_AP1p_CrGES-mVenus* RICE plasmid that contain native *P. tricornutum* genomic regions, such as promoters or terminators, the *pUC19_AP1p_CrGES-mVenus* RICE plasmid sequence was aligned against *P. tricornutum* genome using BLAST (EnsemblProtists, ASM15095v2). Initial hits that only aligned to those regions of *pUC19_AP1p_CrGES-mVenus* that are native to *P. tricornutum* were filtered out as false-positive hits using custom awk commands. The resulting true-positive hits were manually checked to identify the chromosomes of the integration sites. For detailed analyses all reads were mapped to each of the matching chromosomes using bwa v0.7.15 (Li and Durbin, 2009). The resulting sam files were sorted, converted to bam-format and indexed using samtools 1.3.1 (Li et al., 2009) and potential integration sites were manually checked using the Integrative Genomics Viewer v2.4.16 (Robinson et al., 2011). Further analysis for ambiguous hits was performed using the Artemis Comparison Tool (ACT) v13.0.0 (Carver et al., 2005).

## Results and Discussion

Terpenoid engineering in *P. tricornutum* has only recently been reported (D'Adamo et al., 2018; Fabris et al., 2020) and consequently, there is limited prior knowledge to inform metabolic engineering strategies in diatoms to obtain elevated terpenoid production. Recently, we demonstrated that extrachromosomal expression (EE) can be used to efficiently express the fusion protein CrGES-mVenus in *P. tricornutum* cytosol to produce up to 0.21 μg/10^7^ cells (0.309 mg/L) geraniol following bacterial conjugation (Fabris et al., 2020). EE of transgenes is not subject to position effect (Karas et al., 2015a) and could therefore provide highly reproducible, consistent, and controllable expression, which is a basic requisite for synthetic biology. In contrast, randomly integrated chromosomal expression (RICE) can result in genetically dissimilar transformants and consequently varied transgene expression among them. However, diatom phenotypes derived from EE and RICE have not been systematically parameterised. Because little is known regarding the mechanisms and effects following EE and RICE of transgenes, it is unclear how these different engineering strategies will compare regarding the expression of *CrGES-mVenus*, and consequently, heterologous geraniol production. Therefore, we analysed the phenotypes of EE and RICE of *Ap1_CrGES-mVenus* in *P. tricornutum* cell lines both at the expression level and in terms of geraniol yield.

Two identical DNA expression cassettes of *AP1p_CrGES-mVenus* and *AP1p_mVenus* as control (Fabris et al., 2020) were cloned either into an pPtPBR11 episome (Diner et al., 2016) or a pUC19 plasmid (Norrander et al., 1983), and delivered either by bacterial conjugation or DNA-coated particle bombardment, respectively, in order to create EE and RICE *P. tricornutum* transformant libraries. Both *AP1p_CrGES-mVenus* constructs contained the *CrGES* gene fused at the carboxyl-terminus to a mVenus yellow fluorescent protein (YFP) (Kremers et al., 2006) for rapidly screening the cell lines by flow cytometry. The *CrGES-mVenus* fusion gene was driven by the *P. tricornutum* native alkaline phosphatase (*AP1, Phatr3_J49678*) promoter (hereafter *AP1p*), which is induced in low phosphate conditions for controllable expression (Lin et al., 2017).

Upon transformation of *P. tricornutum* with the episomes and plasmids described above, the resulting antibiotic resistant cell lines were used to create four transgenic diatom libraries. Cell lines transformed with *pPtPBR11_AP1p_CrGES-mVenus* and *pPtPBR11_AP1p_mVenus* for EE were denoted as EE_GmV and EE_mV, respectively. Likewise, cell lines transformed with *pUC19_AP1p_CrGES-mVenus* and *pUC19_AP1p_mVenus* for RICE were denoted as RICE_GmV and RICE_mV, respectively.

### EE Transformants Demonstrate Consistent mVenus Fluorescence, While RICE Transformants Demonstrate Higher, but More Variable Signals

In order to compare transgene expression across all four transgenic libraries (EE_GmV, EE_mV, RICE_GmV, and RICE_mV), we required a high-throughput screening strategy to quantify the relative heterologous protein production. We used flow cytometry to rapidly evaluate the CrGES-mVenus expression in an unprecedented number of transformants to identify unique features among and within cell lines generated by EE and RICE. This strategy enabled us to confirm the correct expression of the fusion protein, as well as quantify its relative abundance, offering a proxy for identifying high-expressing transformant variants from the low expressing or silenced variants (Sheff and Thorn, 2004; Delvigne et al., 2014). It is generally accepted that RICE transformants will exhibit different levels of heterologous protein production from each other (Hallmann, 2007; Jeon et al., 2017; Tanwar et al., 2018). Conversely, EE exconjugants theoretically offer more consistent levels of expression (Karas et al., 2015a). However, this has never been shown over a large scale of transformants or through direct comparison. Therefore, it is not known to what extent the expression of heterologous protein can vary across EE exconjugants or RICE transformants.

**Figure 1:**
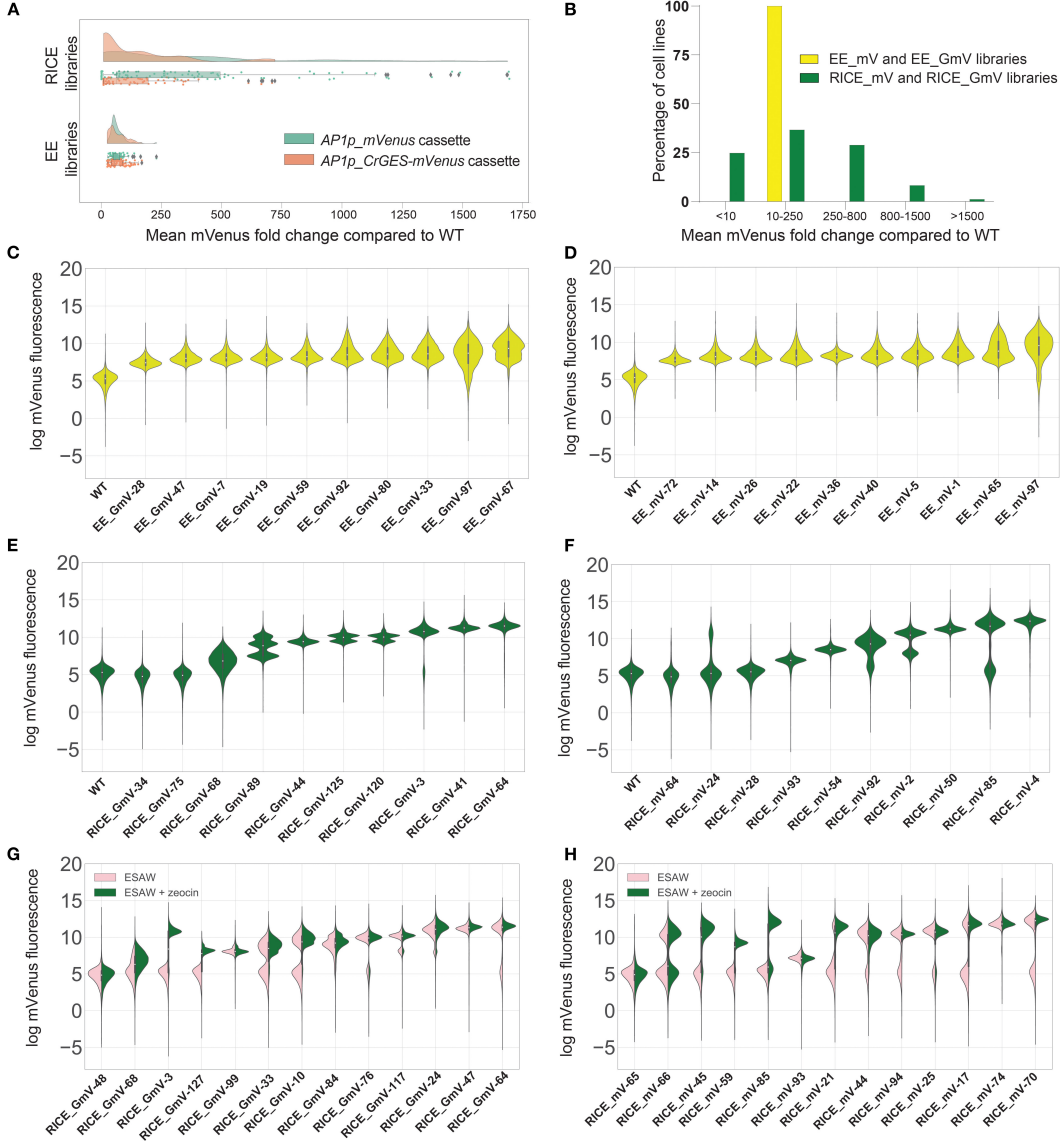
mVenus fluorescence intensities of transgenic *P. tricornutum* extrachromosomal expression (EE) and randomly integrated chromosomal expression (RICE) libraries. **(A)** Fold change of mean mVenus fluorescence of RICE_GmV and RICE_mV transformant libraries and EE_GmV and EE_mV libraries compared to wild type auto-fluorecence. Peach indicates CrGES-mVenus transgenic cell lines and teal indicates mVenus transgenic cell lines. Statistical comparisons were made using Kruskal-Wallis non-parametric ANOVA and Dunn's *post-hoc* test. For EE_GmV library *n* = 96 cell lines total, EE_mV library *n* = 96 cell lines total, RICE_GmV library *n* = 74 cell lines total and RICE_mV library *n* = 95 cell lines total. **(B)** Percentage of pooled RICE libraries (green) compared to percentage of pooled EE libraries (yellow) binned according to mean mVenus fluorescence fold change. **(C–F)** Violin plots indicate the per cell mVenus fluorescence intensity of ten representative cell lines for each library **(C)** EE_GmV **(D)** EE_mV **(E)** RICE_GmV **(F)** RICE_mV, ranked from lowest to mean mVenus expression (*n* = 20,000 cells for each cell line). **(G,H)** Representative cell lines from transgene stability analysis for **(G)** RICE_GmV and **(H)** RICE_mV libraries. Pink indicates selection free growth conditions, green indicates zeocin selection growth conditions, cell lines are ranked by mean mVenus intensity (*n* = 20,000 cells for each cell line).

Single colonies from each of the four libraries were screened according to mVenus fluorescence to evaluate differences in transgene expression. Our results demonstrate that, as predicted and previously reported on a smaller sample (Fabris et al., 2020), EE results in consistent transgene expression among exconjugants. The mean mVenus fluorescence fold change of all EE_GmV lines and all EE_mV lines was 58.50- and 57.73- fold, respectively, when compared to wild type (WT) auto-fluorescence and were not significantly different from each other (*p* > 0.9999) (Figure 1A). This suggested that construct size and complexity—at least of this degree—does not affect expression, and confirms that EE might not be affected by variable transgene silencing typically associated with position effect. For EE_GmV strains, the DNA construct contained the fusion gene geraniol synthase and mVenus (total construct size of 10,849 bp); whereas EE_mV strains were transformed with the mVenus containing DNA (total construct size 9,082 bp). Within each EE library, there was also little variation among EE_GmV lines (SEM = 3.54) and EE_mV lines (SEM = 2.95) (Figure 1A). These results indicate that EE transformants are highly similar. Interestingly, every EE transformant analysed, across both EE_GmV and EE_mV libraries, showed a mean mVenus fluorescence greater than WT auto-fluorescence (Figure 1B). This shows that EE is highly efficient and reliable in generating cell lines that express transgenes and does not require extensive screening, as is required for RICE engineering strategies.

Unlike EE, RICE is subject to position effects and is therefore expected to result in transformants with variable transgene expression. We confirmed this in both RICE_GmV and RICE_mV libraries, in which transformants showed highly variable mVenus fluorescence intensities (Figure 1A). For example, the mean mVenus fluorescence in RICE_GmV lines ranged between 0.04 and 719.20- fold change (SEM = 21.37) and RICE_mV lines ranged between 0.40 and 1695.00- fold change (SEM = 43.22). Furthermore, the RICE_GmV and RICE_mV libraries were significantly different from each other (*p* < 0.0001), demonstrating mean mVenus fold changes of 137.80- and 368.60-fold, respectively. Together these results suggested that when transgenes are integrated randomly in to the genome, features of the transgene, such as size and complexity, may affect its expression. It is plausible that gene silencing plays a role, as mVenus is present as a large fusion protein in the RICE_GmV library, whereas it is a smaller, free fluorescent protein in the RICE_mV library.

To further evaluate the heterogeneity of mVenus fluorescence across RICE libraries, we arbitrarily binned transformants based on the vast spread of mean mVenus fluorescence profiles recorded. We generated five groups comprising of <10-, 10- to 250-, 250- to 800-, 800- to 1,500-, and >1,500-fold change in mVenus fluorescence compared to wild type auto-fluorescence. In the RICE_GmV and RICE_mV libraries (Figure 1B), ~25% of transformants showed <10-fold mean mVenus than wild type auto-fluorescence (Figure 1B). Biolistic bombardment is expected to result in random fragmentation of plasmid DNA (Hopes et al., 2016). This could theoretically result in antibiotic resistant transformants that contain the selection cassette without the intact *AP1p_CrGES-mVenus* transcriptional unit, possibly resulting in antibiotic resistant transformants with fluorescence profiles indistinguishable from WT auto-fluorescence. Additionally, it is plausible that transformants associated with such low mean mVenus signals might have integrated the *CrGES-mVenus* expression cassette at transcriptionally repressed genomic loci, or in arrangements that may have triggered gene silencing (Kim et al., 2015). About 37% of RICE transformants and 100% of EE exconjugants demonstrated mean mVenus fluorescence 10–250-fold greater than WT auto-fluorescence. A further 28% of RICE transformants showed a 250–800-fold increase. Only 7% showed an 800- to 1,500-fold increase, and 1% (corresponding to 2 cell lines both from RICE_mV library) reached a remarkable 1,500-fold increase in fluorescence compared to WT auto-fluorescence.

Together, these results demonstrate that transformants generated by RICE require extensive screening at the protein expression level, as up to a quarter can show no to low expression. Interestingly, RICE transformants were able to demonstrate exceptionally higher maximum transgene expression compared to EE using the same transgene cassette design. This warrants further investigation into these transgenic genomes, as there may be aspects of chromosome-integration that could be useful, particularly with regard to multi-generation transgene stability (Kohli and Christou, 2008). Although this high RICE-related expression could be advantageous for simple transgenic constructs, our results show that it would not be suitable for testing larger, more complex assemblies especially without reporter genes. Instead, the high, virtually size-independent consistency of phenotypes associated with EE offer a more suitable platform for applications involving multi-gene constructs, with the advantage of not requiring large scale screening.

### Clonal Variegation Is Broadly Distributed in EE but Discretely Defined in RICE

After having determined marked differences in the expression profile between EE and RICE libraries, we exploited the resolution of high-throughput flow cytometry to investigate the population composition within each cell line of EE and RICE libraries. In doing so, we identified relevant variations in the distributions of mVenus fluorescence within individual cell lines, known as cell mosaicism or variegation (Kaufman et al., 2008). Most EE transformants showed a relatively homogenous distribution of mVenus abundance within each cell line, such as that of EE_GmV-28,−47,−7 and−19, and EE_mV-72,−14 and−36 (Figures 1C,D). However, some showed increasingly diverse mVenus distribution profiles within individual cell lines, such as EE_GmV-92,−80,−33,−97, and−67 and EE_mV-22,−40,−5,−1,−65, and−97. Intriguingly, these cell lines tended to show higher mean mVenus abundance (Supplementary Figure 1). This suggested that EE transformants which exhibited higher mean mVenus signals were composed of cells that were highly dissimilar from each other in a more continuous, non-discrete manner. This observation raises important questions about the dynamics of EE in diatoms; namely, how are episomal copies maintained within each cell, and how dynamic is episomal copy number and segregation across individual cells at different stages of the life cycle? Such mechanisms have been uncovered in other eukaryotic, non-microalgal species. For example, maintenance of viral episomes in mammalian cells and plasmids in *S. cerevisiae* have been attributed to chromosome tethering and hitchhiking mechanisms (Ghosh et al., 2007; Liu et al., 2008; McBride, 2008; Sau et al., 2019). In yeast synthetic biology research, episomal DNA sequences have been characterised based on traits including transformation efficiency, copy number, transgene expression and plasmid stability of clonal populations (Bouton and Smith, 1986; Nakamura et al., 2018; Gu et al., 2019). For example, Nakamura et al. (2018) tested various episome-regulation sequences in *Pichia pastoris* transgenic strains expressing EGFP extrachromosomally. They reported that strains containing the autonomously replicating sequence (ARS) without a centromeric region (CEN) showed broad fluorescence profiles similar to those that we report here, whereby cells within a single clonal population show a wide spread of EGFP fluorescence. However, when combined with centromeric region (CEN2), they reported a more discrete distribution of high EGFP fluorescence profiles that were more consistent with our RICE transformants. This was likely due to a strong bias for the mother cell over the daughter cells during cell division (Gehlen et al., 2011). While the pPtPBR11 plasmid used in this study contains CEN region (Diner et al., 2016), it is still not yet known how such features contribute to episome expression in diatoms (Karas et al., 2015a). Such factors could influence this cell-to-cell phenotypic heterogeneity, but for unknown reasons, this seemed to become more prominent at higher mean mVenus fluorescence.

Overall, RICE transformants demonstrated homogeneous fluorescence distribution profiles within individual cell lines, such as RICE_GmV-44,−41, and−64 and RICE_mV-93,−54,−50, and−4 (Figures 1E,F). Other RICE transformants also demonstrated heterogeneous mean mVenus profiles, but as numerous discrete populations within single cell lines (Figures 1E,F). For example, RICE_GmV-125,−120 and−3 and RICE_mV-24,−92,−2, and−85 all were composed of two unique populations of mVenus fluorescence distribution (Figures 1E,F). Transformant RICE_GmV-89 even showed three populations (Figure 1E). These results demonstrate that individual cells within a clonal transformant RICE cell line, generally assumed to have identical phenotypes, can be highly heterogeneous with regard to transgene expression, but that this heterogeneity can be distributed into unique, discrete populations. This is a major difference with the highly heterogeneous EE cell lines, which were instead characterized by a wide distribution of heterogeneity within the population, although RICE_GmV-68 transformant also followed this distribution.

### RICE Cell Lines Exhibit Dramatically Varied Stability That Does Not Correlate to *CrGES-mVenus* Expression

Given the extremely high outliers, we investigated the stability of the RICE libraries and how this related to expression level. Random chromosomal integration can result in stable maintenance and expression of transgenes, even in absence of selective pressure. Transformants that looked indistinguishable from wild type auto-fluorescence in the selective treatment did not change when selective pressure was removed (RICE_GmV-48 and RICE_mV-65, Figures 1G,H). We also identified RICE transformants that did not retain their mVenus fluorescence in absence of selective pressure (Figures 1G,H). For example, RICE_GmV-3 and−127 and RICE_mV-45,−59,−85 (Figures 1G,H, respectively) demonstrated a complete reduction in mVenus fluorescence when cultured in the absence of zeocin that was indistinguishable from wild type auto-fluorescence. Without selective pressure, cells that have silenced their resistance transgene (and by proxy, the transgene of interest) can outcompete and take over the culture due to the disadvantages of reduced energy and resource investment associated with transgene expression.

Interestingly, transgene expression level did not correlate to transgene stability, as seen in RICE_GmV-127 and−99, which showed similar expression levels with selection, but only−127 lost mVenus fluorescence when selection was removed. Likewise, RICE_GmV-3 and−127 cell lines show dissimilar mVenus signals in presence of selection but lost signal completely in selection-free conditions (Figures 1G,H). This suggested that there may be transgene integration events or arrangements that facilitate stable transgene expression and highlight the importance of designing screening procedures based on stability not only on transgene expression. Potential mechanisms of action include progressive transcriptional silencing via DNA methylation or histone modification, including *de novo* DNA methylation triggered by transgene recognition (Kohli et al., 2010); and posttranscriptional silencing known as RNA interference (Meyer, 1995; León-Bañares et al., 2004; Cerutti et al., 2011; Doron et al., 2016). Epigenetic silencing of nuclear-integrated exogenous DNA have been attributed to defense mechanisms against viruses and transposable elements in plants (Rajeevkumar et al., 2015) and mammalian cells (Alhaji et al., 2019) alike. Silencing mechanisms, and indeed transgene regulation mechanisms yet to be identified, can influence daughter cells from the same original clonal population differently (Kaufman et al., 2008). In fact, it is not known how stable randomly integrated exogenous DNA fragments are once they have been integrated, or how these insertions are genetically maintained over time.

In other RICE transformants, such as RICE_GmV-68,−33 and−10 and RICE_mV-17 and−70, we detected a reduction in mVenus abundance in absence of selection. Here, a distinct population of cells within each transformant showed signals similar to those in presence of selection, as well as a secondary population of noticeably lower mVenus fluorescence (Figures 1G,H). Other lines showed only a very small reduction in expression, such as RICE_GmV-76,−117, and−64 and RICE_mV-44,−94, and−25. Finally, we were able to identify some RICE transformants that maintained mVenus signals both in presence and absence of selective pressure, namely transformants RICE_GmV-99,−84,−24, and−47 and RICE_mV-66,−93, and−74 (Figures 1G,H). Some of these transformants also demonstrated comparatively high mVenus fluorescence abundance, particularly RICE_GmV-41,−47 and RICE_mV-74 (Supplementary Figure 1). This suggested that they might contain integration events or arrangements that bypass silencing mechanisms. These transformants could provide empirical evidence for putative safe-harbour loci, which have been previously verified in various other organisms including mammalian cell lines (Lee et al., 2015; Cheng et al., 2016; Papapetrou and Schambach, 2016; Salsman and Dellaire, 2016), rice (Cantos et al., 2014) and cyanobacteria (Bentley et al., 2014; Pinto et al., 2015).

Together, these results once again highlight that RICE is not suitable for more complex synthetic biology and that efforts to move toward next-generation genetic engineering strategies is crucial. High expressing RICE transformants can be unstable, as well as contain unknown genomic disturbances and mutations due to damage to the genome itself, as demonstrated in rice and maize (Liu et al., 2018). However, a better understanding of high transgene expression in RICE transformants may reveal aspects of exogenous DNA integration that would be useful for targeted insertion strategies.

### Long-Read Whole-Genome Sequencing Reveals No Chromosomal Integration of Episomal DNA, Whereas Biolistic Bombardment Caused Exogenous DNA to Integrate at Unique Chromosomal Loci

To date, transgenic genomic research has been restricted by prohibitive costs of whole-genome sequencing and limited techniques that only reveal certain aspects of random integration events (Scaife and Smith, 2016; Jeon et al., 2017). In diatoms, such strategies include Southern blotting, which showed 1–10 transgene copies of foreign DNA integrated into the genome (Falciatore et al., 1999); quantitative polymerase chain reaction (qPCR), which revealed that copy number was relatively consistent between transformants variants (average of three) (D'Adamo et al., 2018); and thermal asymmetric interlaced PCR (TAIL-PCR), which revealed integration loci of exogenous DNA (Johansson et al., 2019). Similarly, the recently developed method of EE has been shown to require no transgene integration via episome recovery experiments (Karas et al., 2015a; Diner et al., 2016). However, it is not yet known if integration does occur alongside extrachromosomal maintenance of episomes. Consequently, there is no knowledge of RICE or EE transgenic microalgal genomes with regard to exogenous DNA integration arrangements, the frequency of integration events throughout the genome, or any precise genomic integration loci. Answering some of these knowledge gaps is required for advancing synthetic biology design, progressing next-generation engineering tools –such as possible safe harbour loci to target–, and providing better understanding of the transgenic genome architecture, particularly regions associated with high transgene expression.

Developments in long-read sequencing technologies, namely Oxford Nanopore and PacBio, have allowed more continuous genome assemblies which can be done in real-time in the lab (Jain et al., 2018). To date, these technologies are mostly used for metagenomics analyses (Robertsen et al., 2016; Pinder et al., 2019) or sequencing new, non-model species (Davis et al., 2016; Fournier et al., 2017). Herein, we applied Oxford Nanopore sequencing to interrogate bacteria-conjugated and biolistic-bombarded transgenic *P. tricornutum* cell lines to explore integrated transgene arrangements, integration locations, and associated genetic architecture, as has been recently done in *Arabidopsis thaliana* and mouse models (Jupe et al., 2019; Nicholls et al., 2019). Given the phenotypic consistency between EE lines, we analysed a single EE line, EE_GmV-97, and assessed all the reads for alignment to the *pPtPBR11_AP1p_CrGES-mVenus* episome DNA. For RICE lines, we analysed two lines that showed high stability and mVenus fluorescence, RICE_GmV-41 and−47. These cell lines showed very similar transgene expression profiles and stabilities. Therefore, we aimed to identify differences or similarities regarding their transgenes at the genome-wide scale. These RICE reads were assessed for alignment to the RICE plasmid *pUC19_AP1p_CrGES-mVenus*. All EE and RICE reads with hits to their respective exogenous DNA constructs were then aligned to the wild type *P. tricornutum* genome in order to identify genomic integration events. The results of the Oxford Nanopore sequencing analysis are summarised in Table 1. For each cell line, we sequenced over 250 million nucleotides, resulting in genome coverage of five to nine times, with a probability >99% that the complete genome of each cell line was covered (Clarke and Carbon, 1976).

**Table 1:** Summarised details of MinION sequencing of EE and RICE transformants.

	**EE_ GmV-97**	**RICE_GmV-41_A**	**RICE_GmV-41_B**	**RICE_GmV-47_A**	**RICE_GmV-47_B**
Nucleotides (total)	203,640,859	254,953,905	279,294,842	257,611,430	325,110,058
Reads (total)	26,455	37,373	31,581	18,525	20,153
Average read length	7,697.63	6,822	8,843.60	13,906	16,132.09
Coverage estimated	~5.8x	~ 7.2x	~7.9x	~ 7.3x	~9.2x
Total reads aligning to RICE plasmid	26	248	210	157	151
Reads aligning to RICE plasmid and genome on both borders	0	1	0	0	0
Reads aligning to RICE plasmid and genome on either border	0	18	14	19	27
Reads aligning to RICE plasmid only	26	230	196	138	124

*For RICE_GmV cell lines, the analysis was performed on two biological replicates (A and B)*.

**Figure 2:**
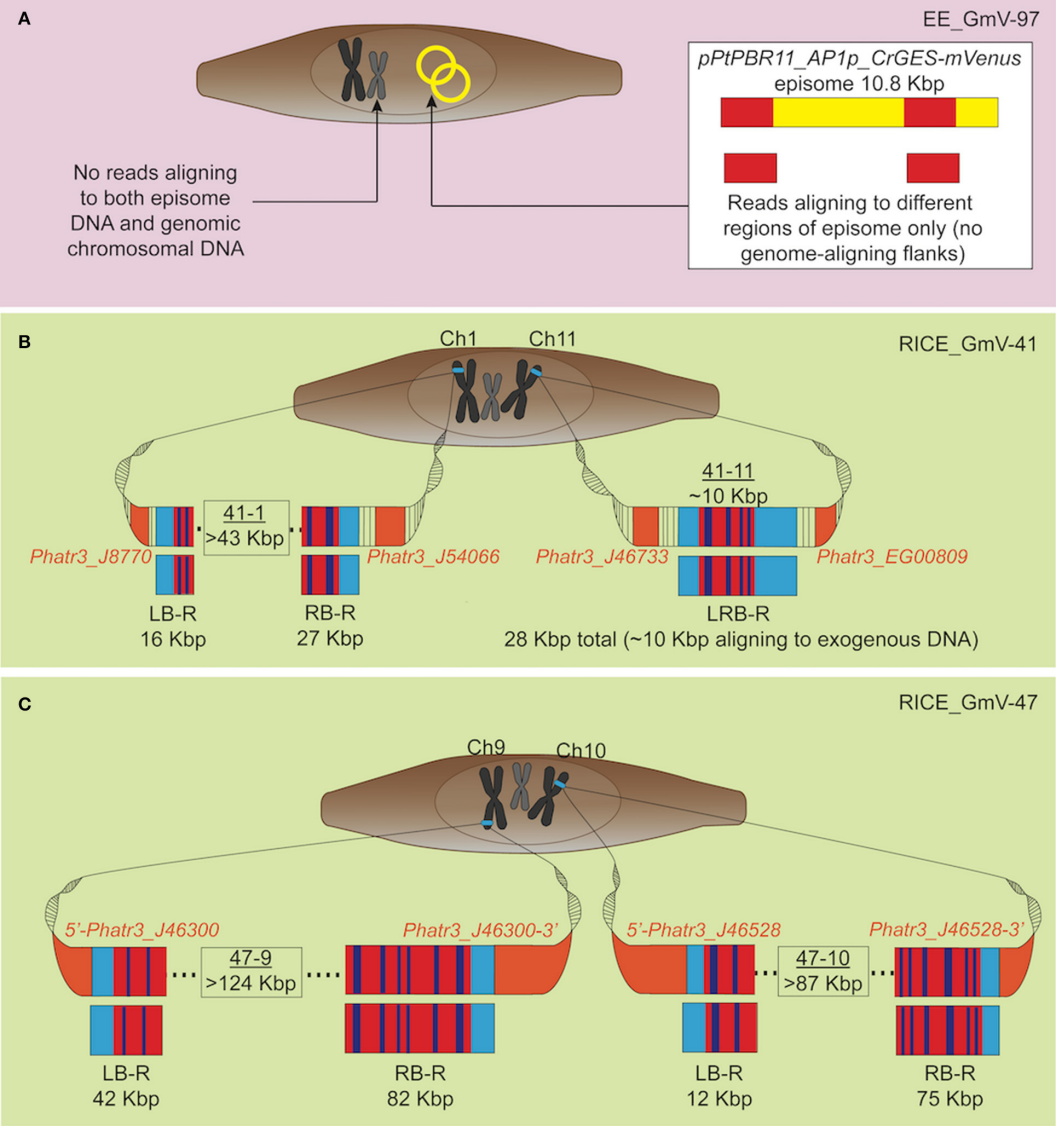
Graphic representation of exogenous DNA constructs in extrachromosomal and chromosomal DNA of the transgenic cell lines, based on long-read sequencing. Only reads aligning to both exogenous DNA and wild type *P. tricornutum* genome, and not those aligning to the genome alone are depicted. **(A)** EE_GmV-97 transformant showed no reads which aligned to both exogenous episomal DNA *pPtPBR11_AP1p_CrGES-mVenus* (yellow), and the reference *P. tricornutum* genome, indicating that no exogenous DNA was integrated into the genome. Instead, some reads showed alignment (red) only to episome DNA, suggesting these reads came from episomal DNA which was extracted and analysed with genomic DNA. **(B)** Transformant RICE_GmV-41 generated by biolistic bombardment showed reads which aligned to both exogenous RICE plasmid, *pUC19_AP1p_CrGES-mVenus*, and the reference *P. tricornutum* genome, indicating a frequency of only two integration islands, 41-1 and 41-11, occurring throughout the whole genome. Island 41-1 occurred on chromosome 1 where the longest left border read (LB-R) and right border read (RB-R) collectively indicated that this island was a minimum of 43 Kbp in size. Island 41-11 occurred on chromosome 11 and was spanned by a single read, left-right border read (LRB-R), which aligned to the reference genome at both left and right borders (light blue), as well as the exogenous RICE plasmid. Red indicates alignment in sense orientation and dark blue indicates alignment in antisense orientation, representing the highly concatenated integration events observed. **(C)** RICE_GmV-47 transformant showed reads which aligned to both exogenous RICE plasmid, *pUC19_AP1p_CrGES-mVenus*, and the reference *P. tricornutum* genome, indicating a frequency of only two integration islands, 47-9 and 47-10, occurring throughout the whole genome. Island 47-9 occurred on chromosome 9 where the longest left border read (LB-R) and right border read (RB-R) collectedly indicate that this island is a minimum of 124 kB in size. Island 47-10 occurred on chromosome 10 where the longest left border read (LB-R) and right border read (RB-R) collectedly indicate that this island is a minimum of 87 Kbp in size.

Our results strongly suggest that no traces of episome were integrated into EE_GmV-97 (Tables 1, 2). Although we identified 26 reads that aligned to the episome, these reads contained no regions which aligned to the *P. tricornutum* genome (Table 1 and Figure 2A), strongly suggesting that these DNA fragments did not get integrated into the nuclear chromosomes. This is the first demonstration that no episomal exogenous DNA is inadvertently integrated into the nuclear genome following bacterial conjugation. Such knowledge is important for identifying any genetic disturbances that may go undetected in exconjugants, progressing knowledge for a better understanding of episomal regulation mechanisms, and for synthetic biology applications with *P. tricornutum* more broadly.

**Table 2:** Summarised details of integration events of EE and RICE transformants.

**Clone**	**Insertion site ID**	**Estimated chromosomal location (bp)**	***In silico* assembly of island complete?**	**Size island (Kbp)**	**Genetic feature at integration site**	**Putative annotation of feature at integration site**	**AA size**	**Upstream features within 1 Kbp**	**Downstream features within 1 Kbp**
EE_GmV-97	None	NA							
RICE_GmV-41	41-1	ch1: 2,477,260	Incomplete.	>43	Intergenic	None	NA	Phatr3_J8770 (protein coding)	Phatr3_J54066 (protein coding)
	41-11	ch11: 316,959–317,016	Complete. (Single read spanned island).	~10	Intergenic	None	NA	Phatr3_J46733 (protein coding)	Phatr3_EG00809 (protein coding)
RICE_GmV-47	47-9	ch9: 865,083–865,119	Incomplete.	>124	Phatr3_J46300 (single exon protein coding gene).	CM000612 Genomic DNA Translation: EEC47937.1	402 aa	NA	Phatr3_J46301 (protein coding)
	47-10	ch10: 609,260–609,276	Incomplete.	>87	Phatr3_J46528 (single exon protein coding gene).	CM000613 Genomic DNA Translation: EEC47687.1	429 aa	NA	NA

*NA, Not applicable*.

In the RICE lines, we identified only two independent integration loci in both RICE_GmV-41 and RICE_GmV-47, respectively (Tables 1, 2; Figures 2B,C). These four sites were detected and confirmed in both biological replicates (Table 2; Supplementary Figure 2). The integration events associated with these four independent loci consisted of concatenations of various fragments of the *pUC19_AP1p_CrGES-mVenus* RICE plasmid (Figures 2B,C). The genomic features and details associated with each of the four integration events are summarised in Table 2.

DNA extracted from each cell line does not come from a single cell, but instead a clonal population, and it is plausible that endogenous genomic regions around the insertion site are not stable (Kohli et al., 1998, 2006). Therefore, it was not possible to resolve every integration event to the single nucleotide level, but only at < 60 bp range. For example, Kohli demonstrated that endogenous DNA concatenations can be assembled prior to or during integration in rice crop species, and suggested recombination could occur even after integration (Kohli et al., 1998, 2006). It is also possible that insertions, deletions, or a combination of both (INDELs) can occur at the borders of an exogenous DNA integration event, driven by non-homologous integration (Shin et al., 2016). Such INDELs would cause reads at this small border region to show no alignment to wild type genome, as seen in RICE_GmV-47 integration event 47-10 (Supplementary Figure 2). Finally, the high sequencing error rate associated with Nanopore sequencing (15%) can also influence integration site determination.

In RICE_GmV-41, fragments of the *pUC19_AP1p_CrGES-mVenus* RICE plasmid were inserted at two unique genomic loci, ch1: 2,477,260 (integration island 41-1) and ch11: 316,959–317,016 (integration island 41-11) (Table 2). Both integration island 41-1 and 41-11 occur at intergenic regions in the genome; however, they are both flanked by predicted protein coding genes (Table 2; Figure 2B). Integration island 41-1 is situated 199 bp downstream of the 3′ end of *Phatr3_J8770* and 479 bp upstream of the 5′ start of *Phatr3_J54066* (Table 2; Figure 2B). *Phatr3_J8770* contains dynamin domains and *Phatr3_J54066* is putatively involved in vesicle trafficking functions according to HMMER (Finn et al., 2011) searches. Integration island 41-11 occurs ~900 bp downstream of the 3′ end of *Phatr3_J46733* and ~100 bp upstream of the 5′ start of *Phatr3_EG00809* (Table 2; Figure 2B). *Phatr3_J46733* contains a transmembrane feature at its C terminus (Uniprot) and a VAD1 Analog of StAR-related lipid transfer domain (VASt) according to HMMER (Finn et al., 2011). *Phatr3_EG00809* showed no predicted functional annotations, nor similarity to known protein domains (Finn et al., 2011).

Neither of these islands disrupted the protein coding regions of these neighbouring genes and we did not detect any growth defective phenotypes for these cell lines. However, the close proximity of the islands to these neighbouring genes means that the integration events may have affected their associated endogenous regulatory regions (Table 2, Figure 2B).

In transformant RICE_GmV-47, two integration events were localised to ch9: 865,083–865,119 (integration island 47-9) and ch10: 609,260–609,276 (integration island 47-10) (Table 2, Figure 2C). Both of these loci harbour predicted single-exon protein coding regions *Phatr3_J46300* and *Phatr3_J46528*, respectively, with no predicted functional annotations, nor similarity to known protein domains (Finn et al., 2011).

Interestingly, all four integration events were contained within unique sites across the entire genome of both cell lines, instead of occurring in a more scattered arrangement at a high number of locations, as has been demonstrated following biolistic bombardment in the plants *Oryza sativa* and *Zea mays* (Liu et al., 2018).

### Biolistic Bombardment Results in Extremely Large Integration Islands Containing Highly Repetitive Arrangements of Exogenous DNA

**Figure 3:**
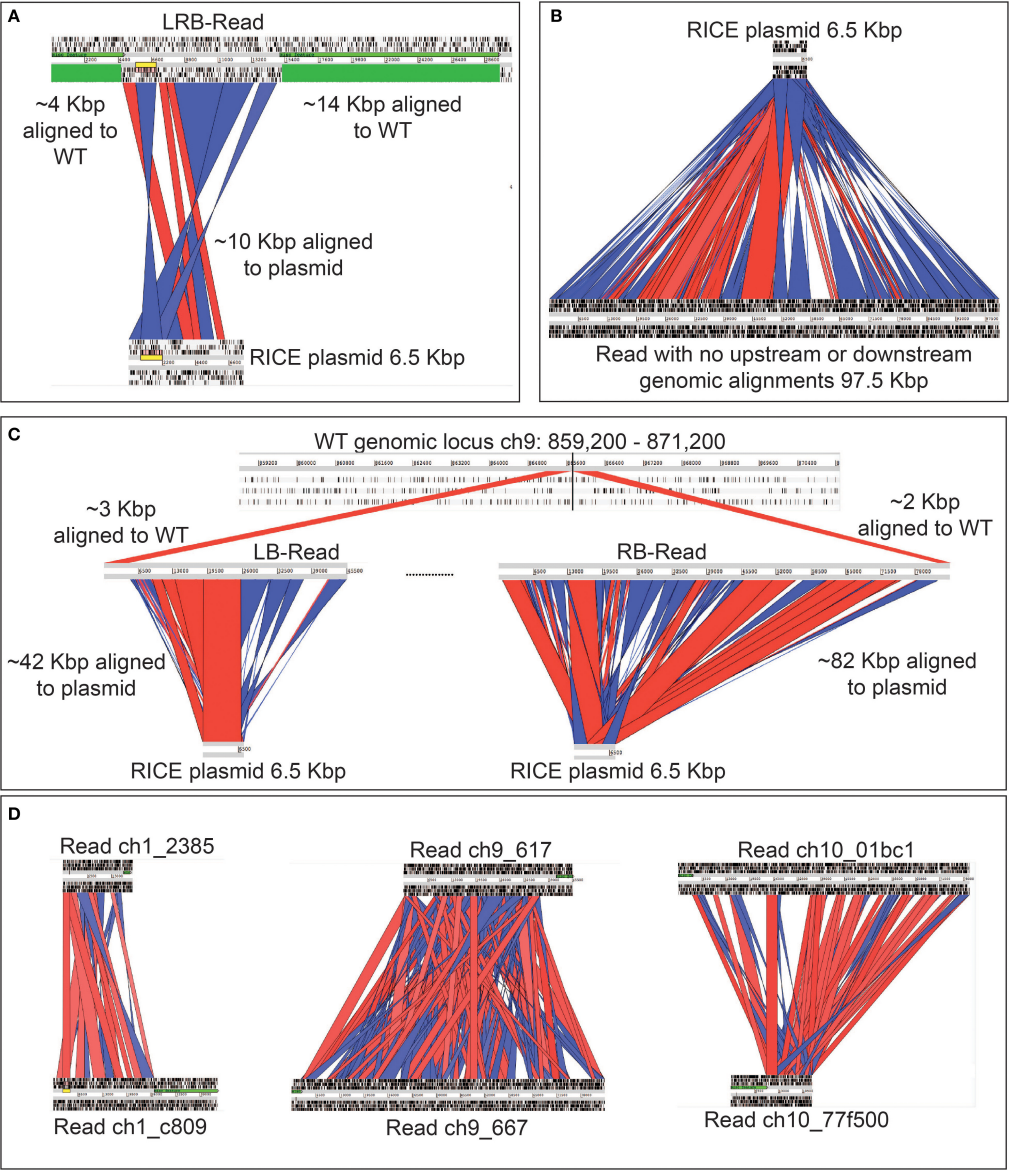
Graphic representation of rearrangements of exogenous DNA in *P. tricornutum* chromosomes, based on long-read sequencing. Red channels show alignment in sense orientation and blue channels show alignment in antisense orientation. Regions that are not highlighted did align to the plasmid, but with below-threshold for hit length of percent identity used for the visualisation, which was performed manually. **(A)** Alignment of a left-right border read (LRB-Read) (top) from integration event 41-11 to RICE plasmid *pUC19_AP1p_CrGES-mVenus* (bottom) and to the wild type *P. tricornutum* genome (green). **(B)** A single 97.5 Kbp read (bottom) with no regions of similarity to the *P. tricornutum* wild type reference genome aligned to the RICE plasmid *pUC19_AP1p_CrGES-mVenus* (top). **(C)** Integration island 47-9 made up by two reads; the left border read (LB-Read) (middle) contains approximately 42 Kbp aligned to the RICE plasmid (bottom) and 3 Kbp aligned to the *P. tricornutum* wild type reference genome (top). The right border read (RB-Read) (middle) contains approximately 82 Kbp of aligned to the RICE plasmid and 2 Kbp aligned to the *P. tricornutum* wild type reference genome. **(D)** Alignments of left and right border reads to each other for integration island 41-1, 47-9, and 47-10. These reads do not align to each other to “close” the integration island, suggesting that some “filler” reads are missing.

Due to the size of the *pUC19_AP1p_CrGES-mVenus* RICE plasmid (6.5 Kbp) and the length of the longer reads we obtained (up to 193.5 Kbp in length), we expected that single reads sequenced using this technology could span the entire integration site. We found this to be true for integration island 41-11, as demonstrated with left-right border read (LRB-R), 28.6 Kbp in length (Figure 2B). This read revealed ~10 Kbp aligned to the RICE plasmid and 4 Kbp upstream and 14 Kbp downstream of the “integration island” aligning to adjacent loci in the reference genome (Figure 3A). This is the first visualisation of a complete integration event in any microalgae and clearly shows both the integration location in the genome as well as the integration event arrangement.

However, this was the only integration event which was detected on a single read. We did not expect that every other integration event would be so large that it would not be detectable within a single long read, but instead only align to one border of the integration island. This is demonstrated by representative reads 47-9_LB-R and 47-9_RB-R that contained a short 5′ flank aligning to ch9: 860,407–865,083 and a 3′ flank aligning to ch9: 865,119–867,673, respectively (Figure 3C). The majority portion of these two reads (42 Kbp for 47-9_RB-R and 82 Kbp for 47-9_RB-R) in fact aligned to RICE plasmid and were found to contain a high frequency of adjacent concatenations of *pUC19_AP1p_CrGES-mVenus* RICE plasmid, in both sense and antisense orientations (Figure 3C). This type of “bordered” configuration was detected around the integration islands 41-1, 47-9, and 47-10.

**Figure 4:**
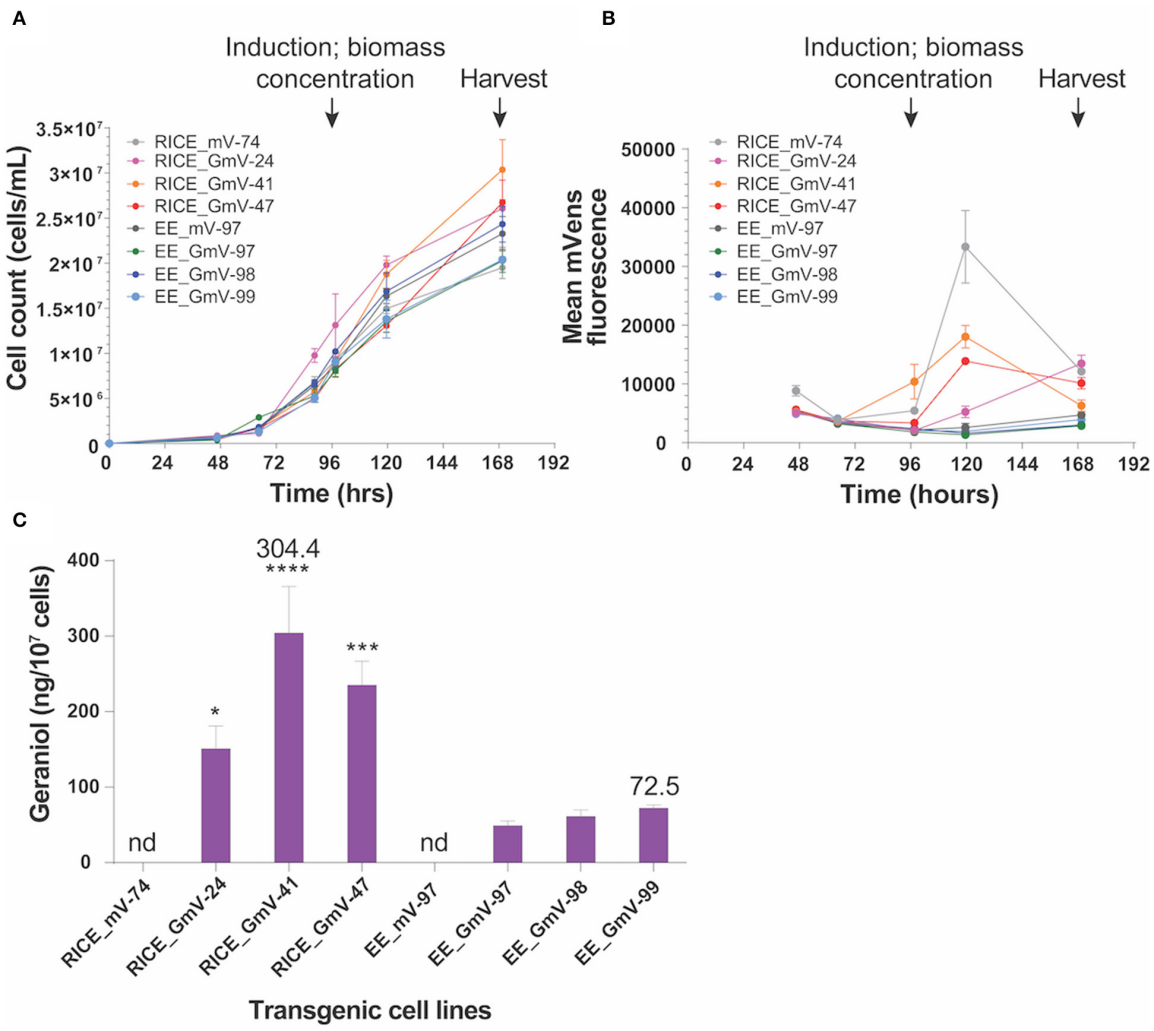
Geraniol production in three selected RICE_GmV and EE_GmV cell lines. *N* = 3, error bars represent SEM, statistical comparisons were made using one-way ANOVA and Tukey's multiple comparisons *post-hoc* test. Significance is demonstrated with asterisks, where ****p ≤ 0.0001; ***p ≤ 0.001; and *p ≤ 0.05. **(A)** Growth curve for all cell lines. **(B)** mVenus fluorescence intensity 24 h after induction. **(C)** geraniol produced after 72 h induction.

The highly repetitive, shuffled structure of the integration islands may be responsible for the high *CrGES-mVenus* expression associated with these cell lines compared to others in this library. Alternatively or in addition to this, it is plausible that either high intact transcriptional unit copy number, or highly repetitive, chimeric promoter and/or terminator arrangements of the *CrGES-mVenus* cassette might act as enhancers for the expression of this construct. Given that we were able to detect mVenus fluorescence and geraniol production (Figures 4B,C), we can infer that some of these fragments contained functional transgene cassettes. However, the ~15% error rate of Nanopore sequencing technology (Jain et al., 2018) and the inability to detect single nucleotide polymorphisms make it near impossible to infer the exact number of functional copies present. Furthermore, it is unclear how stable these large integration islands are, if they are prone to recombination events, or what molecular mechanisms occurred to generate them.

Together, the left and right border reads for integration islands 41-1, 47-9, and 47-9 indicate that these islands are a minimum of 43, 124, and 87 Kbp, respectively. We then looked to assess whether these left and right border reads for these three integration islands aligned to each other to “close” the integration island, but were unable to due to their repetitive nature (Figure 3D).

Interestingly, over 80% of the reads we identified that aligned to the *pUC19_AP1p_CrGES-mVenus* RICE plasmid did so in their entirety (Table 1); i.e., these reads contained no flanks which aligned to the genome at all (Figure 3B). Theoretically, these large reads with no genome-aligning flanks could sit between the left and right border reads of the integration islands. Although we cannot align these highly concatenated reads to each other to confirm this, we speculate that islands 41-1, 47-9, and 47-10 could certainly be hundreds of kilobase pairs in size. Given that RICE_GmV-41 transformant has only two integration events and we knew the size of integration island 41-11 (~10 Kbp) and the sizes of all the “RICE plasmid only” aligning reads together (1,808,244 nt), we can roughly estimate the size of integration island 41-1 through

$$\begin{array}{l} \frac{ni-\left(l_{11}\;*\;c\right)}{c} \end{array}$$

where *ni* is the number of nucleotides in “RICE plasmid only” aligned reads, *l*_11_ is the length of integration island 41-11, and *c* is the estimated coverage. Following this the estimated length of the second integration island 41-1 is ~250 Kpbs. This correlates to 38 hypothetical back-to-back integrations of the full *pUC19_AP1p_CrGES-mVenus* RICE plasmid. Such large hypothetical islands are supported by similar results in transgenic rice and maize lines obtained following biolistic bombardment, in which large integration islands up to 1.6 Mbp in size were reported (Liu et al., 2018). Jupe et al. (2019) also used whole-genome sequencing to elucidate transgene integration structure following *Agrobacterium*-mediated transformation in *Arabidopsis thaliana*. They reported between one and seven integration islands of between 20 and 230 Kbp per strain. While these results are from higher plant species, they confirm that these huge, highly concatenated islands are not specific to biolistic transformation, nor to diatoms.

As previously described, mechanisms involved with RICE are not well-understood but have been investigated in plants (Kohli et al., 2006). Our results highlight a need to explore mechanisms driving the assembly and maintenance of these islands in diatoms, especially given the range of sizes possible that suggest numerous strategies may be at play. This is the first insight into how nuclear integration occurs in diatoms with widespread implications for existing understanding and future studies. Previous short-read sequencing techniques such as targeted gene-walking (Parker et al., 1991), and inverse PCR or TAIL-PCR (Huang et al., 2000; Liu and Chen, 2007; Johansson et al., 2019) have been useful for identifying integration loci, but would not have been able to detect such large integration events of hundreds of kilobase pairs in size, nor would it be able to detect the complex transgene rearrangements (Nicholls et al., 2019) that we unveiled through long-read sequencing. Furthermore, highly concatenated integrations contain many repeated sequences (Figure 3), which nested primers used in TAIL-PCR are able to anneal to. Consequently, PCR strategies would result in many non-specific amplicons and difficulty in determining an unknown integration location. Whilst Southern blotting is a useful approach for determining gene copy number, it does not provide information about the integration site. Hence, our results demonstrate that long-read whole-genome sequencing is an ideal, rapid and affordable approach for determining highly complex transgene integration events.

### RICE *CrGES-mVenus* Transformants Are Associated With Higher Expression and Higher Geraniol Yield

We previously demonstrated that wild type *P. tricornutum* does not naturally produce geraniol, but that it can be efficiently engineered to extrachromosomally express *CrGES-mV* to produce it heterologously (Fabris et al., 2020). In order to determine whether extrachromosomal or chromosomal-integrated expression of the fusion construct *CrGES-mVenus* affected the heterologous production of monoterpenoids, we quantified the amount of geraniol produced in three independent RICE_GmV transformant lines and three EE_GmV exconjugant lines. Using mVenus fluorescence as a proxy for GES expression, we selected six transformants (RICE_GmV-24,−41,−24; and EE_GmV-97,−98 and−99, respectively) based on their mean fluorescence intensity and stability (Supplementary Figure 1). With the aim of enriching the clonal populations associated with higher mean mVenus fluorescence, RICE_mV-74, RICE_GmV-24,−41 and−47 and EE_mV-97, EE_GmV-97,−98 and−99 were induced and sorted based on mVenus fluorescence using fluorescence activated cell sorting (FACS). A preliminary screen of a pooled sample of the top eight RICE_mV transformants was used to define the mVenus positive gate which did not overlap with the wild type control. During FACS, one thousand cells from each transformant that fell into this gate were collected and scaled up. We concluded that cell sorting did not enrich phenotypic populations, and in this specific case did not improve mean mVenus intensity (Supplementary Figure 3).

Geraniol production in transgenic cell lines was evaluated in a bi-phasic, batch fermentation experiment (Fabris et al., 2020). All *CrGES-mVenus* transformants and exconjugants were induced by resuspension in lower volumes (30 ml) of phosphate-free media and showed similar growth to *mVenus* transformant and exconjugant controls prior to induction, indicating no identifiable loss of fitness in *CrGES-mVenus* expressing lines (Figure 4A).

We tracked mVenus fluorescence daily and quantified the accumulated geraniol 72 h after inducing the expression of the *CrGES-mVenus* fusion gene. The RICE_GmV transformants, which showed increased mVenus fluorescence 24 h after induction, produced more geraniol than the episomal exconjugant equivalents (Figures 4B,C). Chromosome-integrated transformant production yields were 150.9 ng/10^7^ cells (0.37 mg/L), 304.4 ng/10^7^ cells (0.89 mg/L) and 235.3 ng/10^7^ cells (0.61 mg/L) for RICE_GmV-24, RICE_GmV-41 and RICE_GmV-47, respectively (Figure 4C). Conversely, non-integrated exconjugants demonstrated consistently lower yields that were 49.1 ng/10^7^ cells (0.10 mg/L), 61.4 ng/10^7^ cells (0.15 mg/L) and 72.5 ng/10^7^ cells (0.15 mg/L) for EE_GmV-97, EE_GmV-98, and EE_GmV-99, respectively (Figure 4C). Neither EE nor RICE mVenus controls showed any detectable geraniol (Figure 4C). These results indicate that mVenus fluorescence is a reliable proxy for geraniol production, as mVenus fluorescence correlated with geraniol yields. Also, the high geraniol yields achieved support the hypothesis that *P. tricornutum* may have an available free pool of cytosolic geranyl diphosphate (GPP), the prenylphosphate precursor that CrGES converts into geraniol (Fabris et al., 2020), and that the heterologous synthesis of this monoterpenoid might not be limited by substrate availability in these settings. In light of these results, strategies involving promoter optimisation and targeted integration—both currently being evaluated in our laboratory—could further increase the geraniol production. Together, our results warrant the development of *P. tricornutum* for enhanced monoterpenoid production and suggest that it would be possible to improve production levels further by optimising *CrGES* expression at the genetic level.

## Conclusions

Within the emerging application of monoterpenoid engineering in diatoms, we set out to generate specific knowledge to inform genetic optimisation strategies, including multi-gene approaches for synthetic biology and pathway engineering. We provided for the first time a comprehensive comparative analysis of two main types of transgene expression in diatoms, the conventional RICE and in the newly developed EE, using large-scale, high-throughput phenotyping, which allowed us to uncover details of these genetic resources between and within cell lines. The genetic differences between EE and RICE were reflected by the varied yields of the relevant monoterpenoid geraniol in a selection of transgenic diatom cell lines expressing a CrGES-mVenus fusion enzyme. The geraniol yield was more than 4-fold higher in the best RICE transformant, reaching the titre of 304.4 ng/10^7^ cells (0.89 mg/L), compared to the best EE exconjugants, reaching 72.47 ng/10^7^ cells (0.15 mg/L). Thus, this work evaluated how previously unexplored genetic strategies can improve heterologous production of geraniol in *P. tricornutum*, in addition to more conventional strategies such as metabolic engineering or bioprocessing. While diatom engineering for terpenoid production has only recently been demonstrated, our results show that *P. tricornutum* is a promising photosynthetic microbial factory (D'Adamo et al., 2018; Fabris et al., 2020).

We report profound differences in the phenotypes of RICE and EE *P. tricornutum* cell lines in terms of expression levels, phenotypic consistency and sub-clonal population composition. Non-integrative episomes are associated with much more consistent phenotypes in the scenarios we tested regarding overexpression and EE exconjugants do not seem to require extensive screening. Furthermore, bacterial conjugation tends to result in more clones in a shorter amount of time than biolistic bombardment. Altogether, these results indicate that EE will be an invaluable resource for genetic parts validation and modular assembly, and even automation of the design-build-test-learn cycle. These aspects of more complex synthetic biology strategies are crucial for heterologous production of high-value products such as monoterpenoids. Our results highlighted the particularly limited knowledge available on key aspects of EE in diatoms. This included stability, copy number and segregation patterns, and episome re-arrangement, which we did not observe but has been reported (Slattery et al., 2018). Such characterisations still need to be addressed to fully exploit EE as a synthetic biology platform in diatoms.

In contrast, RICE cell lines are associated with high variability and overall higher expression levels, and by using large-scale screening it is possible to isolate particularly high expressing lines. These superior diatom cell lines bear highly concatenated arrangements of exogenous DNA, present as vast islands within or nearby predicted protein-coding genes. This raises a concern about this widespread method of generating transgenic diatom cell lines, as disrupting numerous protein coding regions can introduce unknown changes to *P. tricornutum* physiology that may not be easily detected. This is a particularly relevant issue in functional genetics studies involving overexpression, knock-down or knock-out constructs, which are traditionally delivered by biolistics, and randomly integrated in the genome of diatoms. On the contrary, it is not yet known if such large, highly concatenated integration events might be a factor in transgene stability and expression. In such scenario, RICE via biolistic bombardment, might be preferable over EE for obtaining high expressing cell lines. Finally, although it has been shown that high copy number and transgene tandem repeats can cause transcriptional silencing of transgene cassettes in other organisms (Kaufman et al., 2008; Moritz et al., 2016), our findings highlight the need to explore copy number and transgene arrangement optimisation in more detail, as this may well not be the case in *P. tricornutum*.

Whilst it is generally accepted that exogenous DNA delivered by biolistic bombardment randomly integrates in diatom chromosomes, the implications of this may have previously been overlooked, particularly at a time when CRISPR-Cas9 technology is being developed. While there is a general concern in CRISPR research to monitor and prevent off-target cutting by CRISPR-Cas9 itself, our results demonstrate that off-target effects from random integration of exogenous constructs such as vector backbone and DNA-encoded CRISPR-Cas9 components, could be just as much cause for concern. In this way, generating a precise knock-in or knock-out genotype by randomly integrating CRISPR-Cas9 components is suboptimal. As suggested by other works (Sharma et al., 2018; Stukenberg et al., 2018), our findings clearly demonstrate the need to move toward non-integrative alternatives, such as episomal expression (Slattery et al., 2018) and ribonucleoprotein delivery (Serif et al., 2018).

This research also identified putative safe-harbour or neutral loci that could be tested for targeted integration in *P. tricornutum*. Neutral sites in cyanobacteria have been used for targeted integration in metabolic engineering for multigene pathway assembly (Bentley et al., 2014) and dual knock-in knock-out modifications (Li et al., 2016). Synthetic “landing pads” are useful for gene stacking via “domino cloning,” but depend on the knowledge of robust, reliable safe harbour loci prior to being feasibly applied to diatoms (Karas et al., 2015b). While some of the loci we identified harbour predicted protein coding regions, this is not unusual for safe harbours, as seen in human cell lines (e.g., CCR5 and ROSA26 loci) and mouse cell lines (e.g., Rosa26 locus), which all occur within protein coding regions. Furthermore, regions that may currently appear to be intergenic or non-functional may be re-categorised in the future, as more information about “junk DNA,” transcripts without function (TUFs) and unannotated regulatory regions are discovered (Gingeras, 2007).

Finally, to the best of our knowledge, this work reports for the first time the suitability and utility of third generation long-read whole-genome sequencing to reveal the previously unknown nature of chromosomal integration sites, that would not have been feasible with conventional short-read sequencing. Future work investigating trans-genomes, such as low expression RICE cell lines or epigenetic modifications including DNA methylation patterns (Jain et al., 2018; Jupe et al., 2019), would build upon this knowledge to help uncover mechanisms driving transgene integration in diatoms. Such knowledge is important for developing better functional genomics tools including targeted genome editing. Our research primarily aimed at tracking specific, known transgenic constructs in EE and RICE transgenic diatoms cell lines. Long-read whole-genome sequencing technology can also be used to identify changes to the genome independent of an integration event, such as large translocations (Jupe et al., 2019) and deletions (Nicholls et al., 2019), purely due to the disruptive nature of the DNA delivery method. Our work lays the basis for future research efforts specifically focused on these relevant aspects, to investigate the impact of biolistic bombardment itself on genome integrity.

In conclusion, advancing synthetic pathway construction in *P. tricornutum* would ideally combine the reproducibility of EE with the high expression achievable through RICE, which could be achieved by targeted chromosomal integration. This work lays key groundwork for these developments which are crucial for extending knowledge on diatom biology and elevating model species such as *P. tricornutum* as a widely used synthetic biology chassis organism in a broad array of biotechnological applications.

## Data Availability Statement

The datasets generated for this study can be found in NCBI BioProject repository under the ID PRJNA593624.

## Author Contributions

JG designed the study, conducted the experiments, analysed data and wrote the manuscript. TK performed DNA extraction and sequencing, analysed data, and contributed writing the manuscript. RA analysed data and contributed writing the manuscript. UK performed GC-MS analyses and contributed writing the manuscript. PR revised and edited the manuscript. MF designed the study, conducted and supervised the experiments, and contributed writing the manuscript.

## Conflict of Interest

The authors declare that the research was conducted in the absence of any commercial or financial relationships that could be construed as a potential conflict of interest.

## Abbreviations

EE: Episomal expression
RICE: Randomly integrated chromosomal expression.
