# Supplementary material for: Metabolic Engineering Strategies in Diatoms Reveal Unique Phenotypes and Genetic Configurations With Implications for Algal Genetics and Synthetic Biology
Source: Front Bioeng Biotechnol. 2020 Jun 5;8:513. doi: 10.3389/fbioe.2020.00513 (PMC7290003; doi:10.3389/fbioe.2020.00513)
Supplement: Supplementary file 1 [file Data_Sheet_1.docx]

**Metabolic engineering strategies in diatoms reveal unique phenotypes and genetic configurations with implications for algal genetics and synthetic biology**

# Supplementary File 1

# Figure S1: mVenus fluorescence intensities of complete transgenic *P. tricornutum* extrachromosomal expression (EE) and randomly integrated chromosomal expression (RICE) libraries.

# Figure S2: Sequence alignments of MinION reads from each clone, RICE_GmV-41 and RICE_GmV-47, aligned to *P. tricornutum* wild type reference genome, ASM15095v2, at the locations of integration of RICE plasmid DNA, *pUC19_AP1pCrGES-mVenus*.

# Figure S3: mVenus fluorescence population distribution of exconjugants and random integration transformant cell lines used in geraniol analysis sorted by FACS.

#
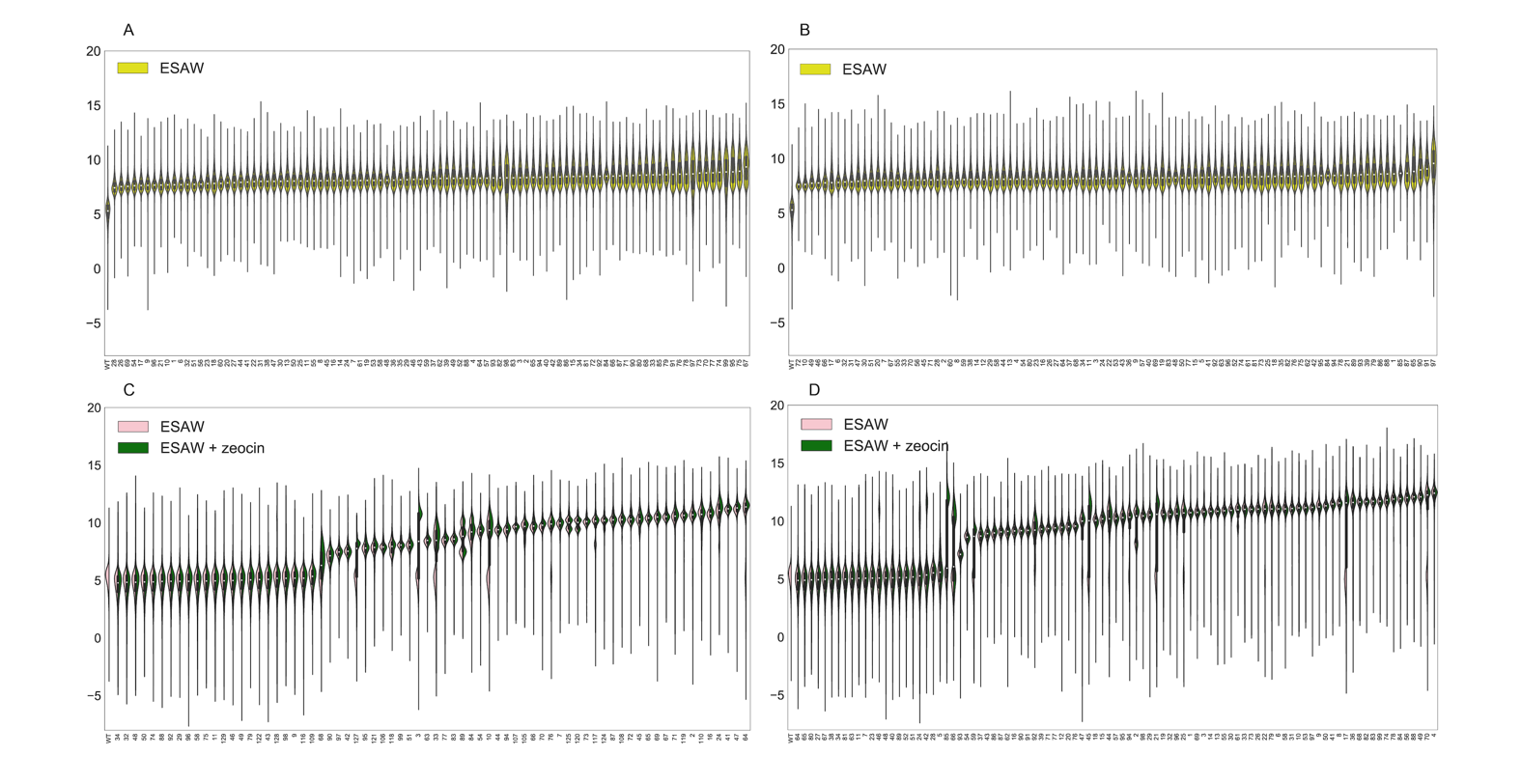


# Suppl. Figure 1 mVenus fluorescence intensities of transgenic *P. tricornutum* extrachromosomal expression (EE) and randomly integrated chromosomal expression (RICE) libraries. Violin plots indicate mVenus fluorescence intensity per cell, of all cell lines for each library. (a) EE_GmV; (b) EE_mV; (c) RICE_GmV; (d) RICE_mV. Pink indicates selection free growth conditions, green and yellow indicates zeocin selection growth conditions, cell lines are ranked by mean mVenus intensity (n = 20,000 cells for each cell line).

#
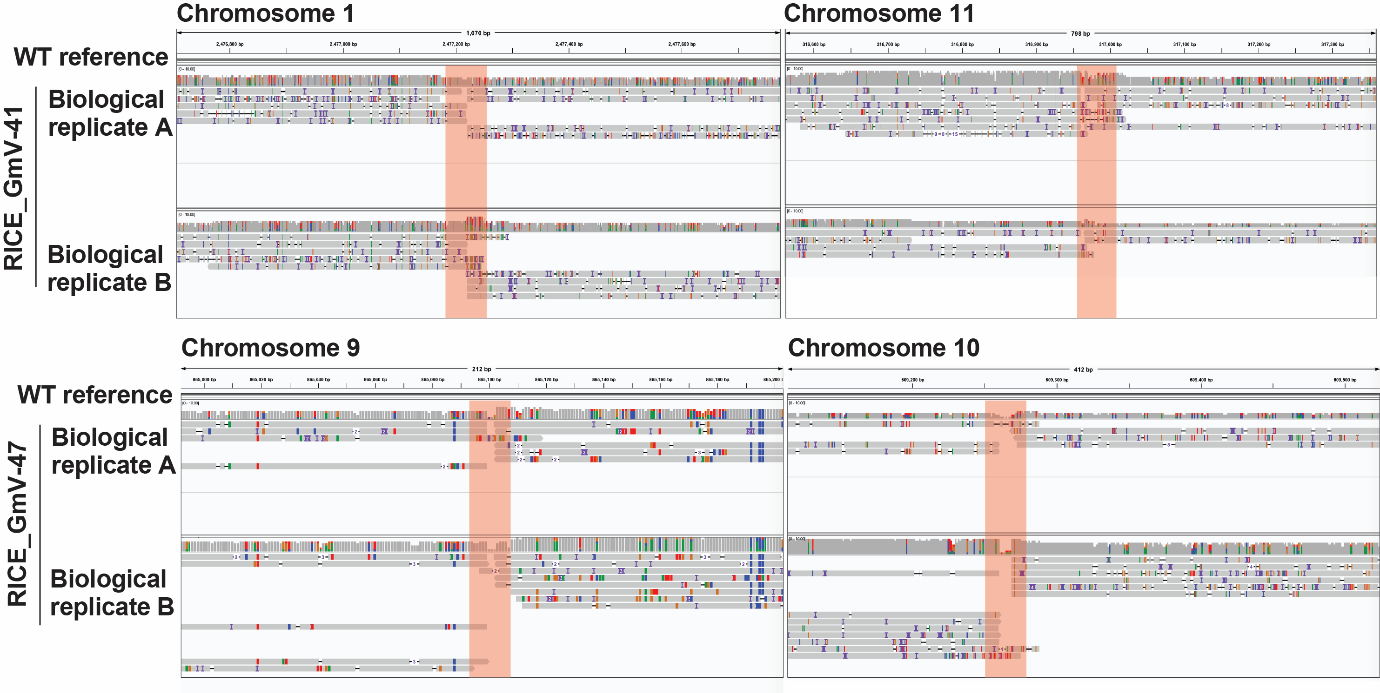


# Suppl. Figure 2 Integration sites in RICE_GmV-41 and RICE_GmV-47 cell lines. Reads obtained from transgenic diatom cell lines that show homology with the *pUC19_AP1pCrGES-mVenus* construct are aligned to *P. tricornutum* wild type reference genome, ASM15095v2. Replicate sequencing experiments per cell line are indicated by either A or B notation. Nucleotides corresponding to the wild type reference genome appears at the top of each frame, with the aligned MinION reads below. Orange bars broadly indicate the regions where integration islands occur.

#
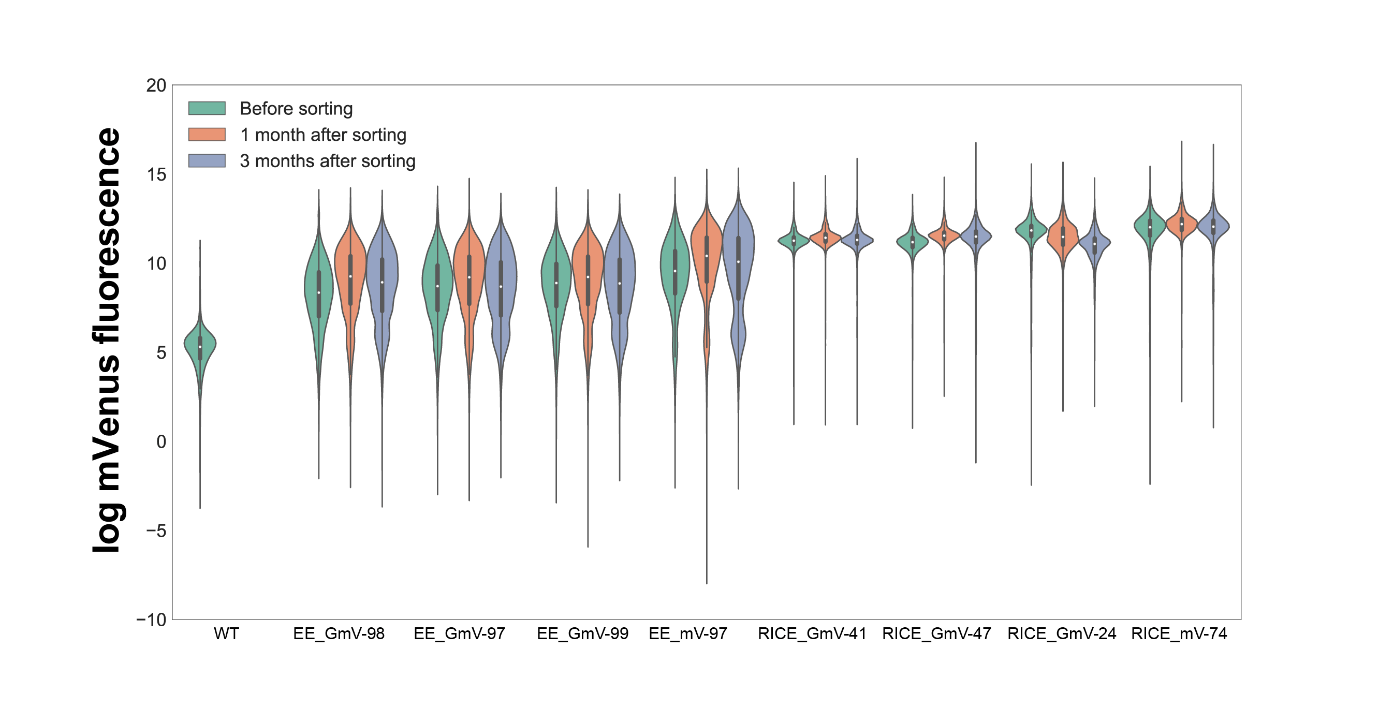


# Suppl. Figure 3 mVenus fluorescence population distribution of exconjugants and random integration transformant cell lines used in geraniol analysis. Population distributions for before, 1 month after, and 3 months after sorting by FACS are shown; n = 20,000 cells.
