# Supplementary material for: Metabolic Engineering Strategies in Diatoms Reveal Unique Phenotypes and Genetic Configurations With Implications for Algal Genetics and Synthetic Biology
Source: Front Bioeng Biotechnol. 2020 Jun 5;8:513. doi: 10.3389/fbioe.2020.00513 (PMC7290003; doi:10.3389/fbioe.2020.00513)
Supplement: Supplementary file 2 [file Data_Sheet_2.docx]

**Metabolic engineering strategies in diatoms reveal unique phenotypes and genetic configurations with implications for algal genetics and synthetic biology**

# Supplementary File 2

LOCUS pPTPBR11_pAP1-Cr 10849 bp DNA circular UNA 30-JUL-2018

DEFINITION A new nucleotide sequence entered manually.

ACCESSION urn.local...v-ba2hx40

VERSION urn.local...v-ba2hx40

KEYWORDS .

SOURCE

ORGANISM .

FEATURES Location/Qualifiers

promoter 3659..4487

/standard_name="AP1p"

ORIGIN

1 acgcggaagt cagcgccctg caccattatg ttccggatct gcatcgcagg atgctgctgg

61 ctaccctgtg gaacacctac atctgtatta acgaagcgct ggcattgacc ctgagtgatt

121 tttctctggt cccgccgcat ccataccgcc agttgtttac cctcacaacg ttccagtaac

181 cgggcatgtt catcatcagt aacccgtatc gtgagcatcc tctctcgttt catcggtatc

241 attaccccca tgaacagaaa tcccccttac acggaggcat cagtgaccaa acaggaaaaa

301 accgccctta acatggcccg ctttatcaga agccagacat taacgcttct ggagaaactc

361 aacgagctgg acgcggatga acaggcagac atctgtgaat cgcttcacga ccacgctgat

421 gagctttacc gcagctgcct cgcgcgtttc ggtgatgacg gtgaaaacct ctgacacatg

481 cagctcccgg agacggtcac agcttgtctg taagcggatg ccgggagcag acaagcccgt

541 cagggcgcgt cagcgggtgt tggcgggtgt cggggcggat cgtcttgcct tgctcgtcgg

601 tgatgtactt caccagctcc gcgaagtcgc tcttcttgat ggagcgcatg gggacgtgct

661 tggcaatcac gcgcaccccc cggccgtttt agcggctaaa aaagtcatgg ctctgccctc

721 gggcggacca cgcccatcat gaccttgcca agctcgtcct gcttctcttc gatcttcgcc

781 agcagggcga ggatcgtggc atcaccgaac cgcgccgtgc gcgggtcgtc ggtgagccag

841 agtttcagca ggccgcccag gcggcccagg tcgccattga tgcgggccag ctcgcggacg

901 tgctcatagt ccacgacgcc cgtgattttg tagccctggc cgacggccag caggtaggcc

961 gacaggctca tgccggccgc cgccgccttt tcctcaatcg ctcttcgttc gtctggaagg

1021 cagtacacct tgataggtgg gctgcccttc ctggttggct tggtttcatc agccatccgc

1081 ttgccctcat ctgttacgcc ggcggtagcc ggccagcctc gcagagcagg attcccgttg

1141 agcaccgcca ggtgcgaata agggacagtg aagaaggaac acccgctcgc gggtgggcct

1201 acttcaccta tcctgcccgg ctgacgccgt tggatacacc aaggaaagtc tacacgaacc

1261 ctttggcaaa atcctgtata tcgtgcgaaa aaggatggat ataccgaaaa aatcgctata

1321 atgaccccga agcagggtta tgcagcggaa gatgccattc gccattcagg ctgcgcaact

1381 gttgggaagg gcgatcggtg cgggcctctt cgctattacg ccagctggcg aaagggggat

1441 gtgctgcaag gcgattaagt tgggtaacgc cagggttttc ccagtcacga cgttgtaaaa

1501 cgacggccag tgaccatgat tacgccaagc tcgaaattaa ccctcactaa agggaacaaa

1561 agctggtacc taacaggatt agtgcaattc gagttgaatc actgggaaaa acattgtctt

1621 cttttttata ttatcatttg cattagtgct gcagtcgtag atacttgttg gttgaaagac

1681 atcagctggg agggactgga ctagcgtttg gtaaggagac atacctgtta acgttggttg

1741 caaaattcca tttcgcgatt tatgttatct gtaaatcctg atttgtctgg aattcttgat

1801 acttccgttt ttttagaggc caatgattag catcggcgat tctcaaaata gcattttcga

1861 catgcggtgc tgatttcata aacatagaca acgcttttac atgtaaaagt aacttgcgga

1921 cttggaacag tgctctgttt ttggtgtgaa cgtaactcag caatatttct gtgctagcaa

1981 ggttttttat gatcgaccga agatctcaaa actccgggtc tttcaactgt ctgactagac

2041 catgttcgta acgtcggaca gcagctttcg ttgtactggt agaatttcta cgtgcgaagc

2101 acgtgtaggc aggttgaacg acgatccctg ccgatggatg gattggcacg cggcggaacg

2161 ctttcgtgat ctacaccacc tggatcttca catatcttcg aaatcgaaaa attaaccaag

2221 tcgacggtat cgataatatt ctagctgagg gtacccatgg ccaagttgac cagtgccgtt

2281 ccggtgctca ccgcgcgcga cgtcgccgga gcggtcgagt tctggaccga ccggctcggg

2341 ttctcccggg acttcgtgga ggacgacttc gccggtgtgg tccgggacga cgtgaccctg

2401 ttcatcagcg cggtccagga ccaggtggtg ccggacaaca ccctggcctg ggtgtgggtg

2461 cgcggcctgg acgagctgta cgccgagtgg tcggaggtcg tgtccacgaa cttccgggac

2521 gcctccgggc cggccatgac cgagatcggc gagcagccgt gggggcggga gttcgccctg

2581 cgcgacccgg ccggcaactg cgtgcacttc gtggccgagg agcaggactg accgacgccg

2641 accaacaccg ccggtccgac gcggcccgac gggtccgagg cctcggagat ctgggcccat

2701 gcggccgcaa caactacctc gactttggct gggacacttt cagtgaggac aagaagcttc

2761 agaagcgtgc tatcgaactc aaccagggac gtgcggcaca aatgggcatc cttgctctca

2821 tggtgcacga acagttggga gtctctatcc ttccttaaaa atttaatttt cattagttgc

2881 agtcactccg ctttggtttc acagtcagga ataacactag ctcgtcttca atcacgtgct

2941 ataaaaataa ttataattta aattttttaa tataaatata taaattaaaa atagaaagta

3001 aaaaaagaaa ttaaagaaaa aatagttttt gttttccgaa gatgtaaaag actctagggg

3061 gatcgccaac aaatactacc ttttaccttg ctcttcctgc tctcaggtat taatgccgaa

3121 ttgtttcatc ttgtctgtgt agaagaccac acacgaaaat cctgtgattt tacattttac

3181 ttatcgttaa tcgaatgtat atctatttaa tctgcttttc ttgtctaata aatatatatg

3241 taaagtacgc tttttgttga aattttttaa acctttgttt attttttttt cttcattccg

3301 taactcttct accttcttta tttactttct aaaatccaaa tacaaaacat aaaaataaat

3361 aaacacagag taaattccca aattattcca tcattaaaag atacgaggcg cgtgtaagtt

3421 acaggcaagc gatcctagta cactctatat ttttttatgc ctcggtaatg attttcattt

3481 tttttttcca cctagcggat gactcttttt ttttcttagc gattggcatt atcacataat

3541 gaattataca ttatataaag taatgtgatt tcttcgaaga atatactaaa aaatgagcag

3601 gcaagataaa cgaaggcaaa gatgacagag cagactctag agtcgacctg cacatatgcc

3661 aaaatttcgt tcacggcagg gattttggcg tcgagatctt catgggacca aaactagcag

3721 gaggattggt ttttcagcat aatagattcg tgtccccgtt tcgatttttc tctgcggccg

3781 tccccaccgg acgctgaaaa atttcaaagc tgaagcattt ggtgaagtcg tacgccaagt

3841 ttcccaccag aaataatgtg gaccaagagg gttaagtgtc gcgcatgtgc gaagaatgcg

3901 ctgggagctg acttcccggc cgactgtaat tagagaagaa catagattaa aaaaagactt

3961 gtcaattcgg aagatacaca agtagacaca gaatactaaa caagaagaga gaagcatgac

4021 tcatcttttt aagcatcata atagatgatt tgcggggcgt ttgtatgagt cagccaagtg

4081 caaatatgtt ttacgtgtca acgaatctta aagatccgtg acaaaaacgc catacaccgt

4141 accatgaaat ggacgctttg ttaacgtaaa gtgtcgaatc ttcagtacac atttttcaag

4201 attcgctaga ttcgacagtg ataaatcatc tttgtcaaaa caatcttcat ggttgtcgaa

4261 cgatatacgg tctatacgtc ctaaatcaga gaaccgttca caaattacaa ttagattctt

4321 tagagaaacc atgaccaggc tcacgtgtac tttcttacaa ttgatgggat cggatgcaag

4381 accgaggaaa agatctcatc tcttgggcgc actgataaag ctcataattt tgtgagttgc

4441 caccaaggat ttttctatcg gcatcctaac attatcggac ctgcaaaact agtatggccg

4501 ctacgatcag taacttgtcg tttttggcta agtcacgcgc attgtctcgc ccttcttcta

4561 gttcgctttc ttggcttgaa cgcccgaaaa cttcgtcaac tatctgtatg tccatgccct

4621 caagttcctc gtctagttca tcttcctcta tgtcccttcc acttgcaaca cctctcatta

4681 aagacaacga atcacttatt aagtttctca gacaaccctt ggttctccct catgaggtag

4741 acgactcgac aaaacgtcga gagttgttgg agagaacccg caaagaattg gagcttaacg

4801 ccgaaaaacc gttggaggca ctcaaaatga tcgacatcat ccagagactc ggactttcgt

4861 atcactttga ggatgatatc aattcaatcc ttacaggctt tagtaacatt tcgagtcaga

4921 cgcacgaaga cctcttgact gctagcctct gcttcagact cttgcgacac aacggccata

4981 agattaatcc agatattttc caaaaattta tggacaataa cggaaagttc aaagattcgc

5041 ttaaggacga cacattgggt atgctctcgc tctacgaagc ttcgtatctc ggagcaaacg

5101 gcgaggagat ccttatggag gctcaagagt ttacaaagac ccaccttaaa aattcgttgc

5161 cagcaatggc tccatctctt tcaaaaaagg ttagccaagc acttgaacag ccccgccacc

5221 gtcgtatgct ccgcctcgag gcaaggcgat ttatcgaaga atatggggcg gagaacgacc

5281 ataatcccga cttgcttgaa ttggccaagc ttgattacaa taaggttcaa agcttgcacc

5341 agatggagct tagtgagatc actcgctggt ggaaacagct cgggttggtc gacaaactta

5401 cgttcgccag ggatcgaccg cttgaatgct ttctctggac cgtagggttg cttccagagc

5461 ccaagtatag tggttgcaga atcgaacttg caaaaaccat tgccattctt cttgtcattg

5521 acgatatttt cgatacgcac ggcacccttg atgaattgtt gctctttacg aatgcgatca

5581 agagatggga ccttgaagcg atggaagatc tcccagagta tatgaggatc tgctatatgg

5641 ccctttacaa tacgacaaac gaaatttgct ataaagtatt gaaggaaaac ggttggtcgg

5701 tccttccgta tcttaaagcg acgtggattg acatgatcga ggggtttatg gttgaagctg

5761 aatggtttaa ctccgattac gtccccaata tggaagaata cgtggagaac ggagtgcgaa

5821 ccgcaggatc ttacatggcg cttgtccact tgtttttcct cattggccaa ggcgtcactg

5881 aggataacgt gaaacttctc attaagccct atccaaagct ttttagcagc tcaggacgta

5941 ttttgcgact ctgggatgat ttgggtacgg ctaaggaaga acaggaacgc ggagatctcg

6001 cttcctcaat ccaacttttt atgagggaga aagagattaa gtcagaagag gagggcagaa

6061 aggggatctt ggagatcatt gagaacctct ggaaagagct taacggtgaa cttgtatacc

6121 gcgaggagat gccgcttgcg atcatcaaaa cggcattcaa tatggcccgt gcttcacagg

6181 tggtttatca acacgaagag gacacttatt ttagttctgt tgacaactat gtaaaagcat

6241 tgtttttcac gccctgcttc atggtgagca agggcgagga gctgttcacc ggggtggtgc

6301 ccatcctggt cgagctggac ggcgacgtaa acggccacaa gttcagcgtg tccggcgagg

6361 gcgagggcga tgccacctac ggcaagctga ccctgaagct gatctgcacc accggcaagc

6421 tgcccgtgcc ctggcccacc ctcgtgacca ccctgggcta cggcctgcag tgcttcgccc

6481 gctaccccga ccacatgaag cagcacgact tcttcaagtc cgccatgccc gaaggctacg

6541 tccaggagcg caccatcttc ttcaaggacg acggcaacta caagacccgc gccgaggtga

6601 agttcgaggg cgacaccctg gtgaaccgca tcgagctgaa gggcatcgac ttcaaggagg

6661 acggcaacat cctggggcac aagctggagt acaactacaa cagccacaac gtctatatca

6721 ccgccgacaa gcagaagaac ggcatcaagg ccaacttcaa gatccgccac aacatcgagg

6781 acggcggcgt gcagctcgcc gaccactacc agcagaacac ccccatcggc gacggccccg

6841 tgctgctgcc cgacaaccac tacctgagct accagtccaa gctgagcaaa gaccccaacg

6901 agaagcgcga tcacatggtc ctgctggagt tcgtgaccgc cgccgggatc actctcggca

6961 tggacgagct gtacaagtaa ctacctcgac tttggctggg acactttcag tgaggacaag

7021 aagcttcaga agcgtgctat cgaactcaac cagggacgtg cggcacaaat gggcatcctt

7081 gctctcatgg tgcacgaaca gttgggagtc tctatccttc cttaaaaatt taattttcat

7141 tagttgcagt cactccgctt tggtttcaca gtcaggaata acactagctc gtcttcatgc

7201 aggcatctct tccgcttcct cgctcactga ctcgctgcgc tcggtcgttc ggctgcggcg

7261 agcggtatca gctcactcaa aggcggtaat acggttatcc acagaatcag gggataacgc

7321 aggaaagaac atgtgagcaa aaggccagca aaaggccagg aaccgtaaaa aggccgcgtt

7381 gctggcgttt ttccataggc tccgcccccc tgacgagcat cacaaaaatc gacgctcaag

7441 tcagaggtgg cgaaacccga caggactata aagataccag gcgtttcccc ctggaagctc

7501 cctcgtgcgc tctcctgttc cgaccctgcc gcttaccgga tacctgtccg cctttctccc

7561 ttcgggaagc gtggcgcttt ctcatagctc acgctgtagg tatctcagtt cggtgtaggt

7621 cgttcgctcc aagctgggct gtgtgcacga accccccgtt cagcccgacc gctgcgcctt

7681 atccggtaac tatcgtcttg agtccaaccc ggtaagacac gacttatcgc cactggcagc

7741 agccactggt aacaggatta gcagagcgag gtatgtaggc ggtgctacag agttcttgaa

7801 gtggtggcct aactacggct acactagaag gacagtattt ggtatctgcg ctctgctgaa

7861 gccagttacc ttcggaaaaa gagttggtag ctcttgatcc ggcaaacaaa ccaccgctgg

7921 tagcggtggt ttttttgttt gcaagcagca gattacgcgc agaaaaaaag gatctcaaga

7981 agatcctttg atcttttcta cggggtctga cgctcagtgg aacgaaaact cacgttaagg

8041 gattttggtc atgagattat caaaaaggat cttcacctag atccttttaa attaaaaatg

8101 aagttttaaa tcaatctaaa gtatatatga gtaaacttgg tctgacagtt accaatgctt

8161 aatcagtgag gcacctatct cagcgatctg tctatttcgt tcatccatag ttgcctgact

8221 ccccgtcgtg tagataacta cgatacggga gggcttacca tctggcccca gtgctgcaat

8281 gataccgcga gacccacgct caccggctcc agatttatca gcaataaacc agccagccgg

8341 aagggccgag cgcagaagtg gtcctgcaac tttatccgcc tccatccagt ctattaattg

8401 ttgccgggaa gctagagtaa gtagttcgcc agttaatagt ttgcgcaacg ttgttgccat

8461 tgctgcaggc atcgtggtgt cacgctcgtc gtttggtatg gcttcattca gctccggttc

8521 ccaacgatca aggcgagtta catgatcccc catgttgtgc aaaaaagcgg ttagctcctt

8581 cggtcctccg atcgttgtca gaagtaagtt ggccgcagtg ttatcactca tggttatggc

8641 agcactgcat aattctctta ctgtcatgcc atccgtaaga tgcttttctg tgactggtga

8701 gtactcaacc aagtcattct gagaatagtg tatgcggcga ccgagttgct cttgcccggc

8761 gtcaacacgg gataataccg cgccacatag cagaacttta aaagtgctca tcattggaaa

8821 acgttcttcg gggcgaaaac tctcaaggat cttaccgctg ttgagatcca gttcgatgta

8881 acccactcgt gcacccaact gatcttcagc atcttttact ttcaccagcg tttctgggtg

8941 agcaaaaaca ggaaggcaaa atgccgcaaa aaagggaata agggcgacac ggaaatgttg

9001 aatactcata ctcttccttt ttcaatatta ttgaagcatt tatcagggtt attgtctcat

9061 gagcggatac atatttgaat gtatttagaa aaataaacaa ataggggttc cgcgcacatt

9121 tccccgaaaa gtgccacctg acgtctaaga aaccattatt atcatgacat taacctataa

9181 aaataggcgt atcacgaggc cctttcgtct tcaagaattc tcatgtttga cagcttatca

9241 tcgataagct ttaatgcggt agtttatcac agttaaattg ctaacgcagt caggcaccgt

9301 gtatgaaatc taacaatgcg ctcatcgtca tcctcggcac cgtcaccctg gatgctgtag

9361 gcataggctt ggttatgccg gtactgccgg gcctcttgcg ggatatcgtc cattccgaca

9421 gcatcgccag tcactatggc gtgctgctag cgctatatgc gttgatgcaa tttctatgcg

9481 cacccgttct cggagcactg tccgaccgct ttggccgccg cccagtcctg ctcgcttcgc

9541 tacttggagc cactatcgac tacgcgatca tggcgaccac acccgtcctg tggatcctct

9601 acgccggacg catcgtggcc ggcatcaccg gcgccacagg tgcggttgct ggcgcctata

9661 tcgccgacat caccgatggg gaagatcggg ctcgccactt cgggctcatg agcgcttgtt

9721 tcggcgtggg tatggtggca ggccccgtgg ccgggggact gttgggcgcc atctccttgc

9781 atgcaccatt ccttgcggcg gcggtgctca acggcctcaa cctactactg ggctgcttcc

9841 taatgcagga gtcgcataag ggagagcgtc gaccgatgcc cttgagagcc ttcaacccag

9901 tcagctcctt ccggtgggcg cggggcatga ctatcgtcgc cgcacttatg actgtcttct

9961 ttatcatgca actcgtagga caggtgccgg cagcgctctg ggtcattttc ggcgaggacc

10021 gctttcgctg gagcgcgacg atgatcggcc tgtcgcttgc ggtattcgga atcttgcacg

10081 ccctcgctca agccttcgtc actggtcccg ccaccaaacg tttcggcgag aagcaggcca

10141 ttatcgccgg catggcggcc gacgcgctgg gctacgtctt gctggcgttc gcgacgcgag

10201 gctggatggc cttccccatt atgattcttc tcgcttccgg cggcatcggg atgcccgcgt

10261 tgcaggccat gctgtccagg caggtagatg acgaccatca gggacagctt caaggatcgc

10321 tcgcggctct taccagccta acttcgatca ttggaccgct gatcgtcacg gcgatttatg

10381 ccgcctcggc gagcacatgg aacgggttgg catggattgt aggcgccgcc ctataccttg

10441 tctgcctccc cgcgttgcgt cgcggtgcat ggagccgggc cacctcgacc tgaatggaag

10501 ccggcggcac ctcgctaacg gattcaccac tccaagaatt ggagccaatc aattcttgcg

10561 gagaactgtg aatgcgcaaa ccaacccttg gcagaacata tccatcgcgt ccgccatctc

10621 cagcagccgc acgcggcgca tctcgggcag cgttgggtcc tggccacggg tgcgcatgat

10681 cgtgctcctg tcgttgagga cccggctagg ctggcggggt tgccttactg gttagcagaa

10741 tgaatcaccg atacgcgagc gaacgtgaag cgactgctgc tgcaaaacgt ctgcgacctg

10801 agcaacaaca tgaatggtct tcggtttccg tgtttcgtaa agtctggaa

//

LOCUS pPTPBR11_pAP1-mV 9082 bp DNA circular UNA 11-DEC-2017

DEFINITION Venus, phosphate starv inducible, swappable.

ACCESSION urn.local...w-ba2hx40

VERSION urn.local...w-ba2hx40

KEYWORDS .

SOURCE Phaeodactlyum tricornutum

ORGANISM Phaeodactlyum tricornutum.

FEATURES Location/Qualifiers

ORIGIN

1 acgcggaagt cagcgccctg caccattatg ttccggatct gcatcgcagg atgctgctgg

61 ctaccctgtg gaacacctac atctgtatta acgaagcgct ggcattgacc ctgagtgatt

121 tttctctggt cccgccgcat ccataccgcc agttgtttac cctcacaacg ttccagtaac

181 cgggcatgtt catcatcagt aacccgtatc gtgagcatcc tctctcgttt catcggtatc

241 attaccccca tgaacagaaa tcccccttac acggaggcat cagtgaccaa acaggaaaaa

301 accgccctta acatggcccg ctttatcaga agccagacat taacgcttct ggagaaactc

361 aacgagctgg acgcggatga acaggcagac atctgtgaat cgcttcacga ccacgctgat

421 gagctttacc gcagctgcct cgcgcgtttc ggtgatgacg gtgaaaacct ctgacacatg

481 cagctcccgg agacggtcac agcttgtctg taagcggatg ccgggagcag acaagcccgt

541 cagggcgcgt cagcgggtgt tggcgggtgt cggggcggat cgtcttgcct tgctcgtcgg

601 tgatgtactt caccagctcc gcgaagtcgc tcttcttgat ggagcgcatg gggacgtgct

661 tggcaatcac gcgcaccccc cggccgtttt agcggctaaa aaagtcatgg ctctgccctc

721 gggcggacca cgcccatcat gaccttgcca agctcgtcct gcttctcttc gatcttcgcc

781 agcagggcga ggatcgtggc atcaccgaac cgcgccgtgc gcgggtcgtc ggtgagccag

841 agtttcagca ggccgcccag gcggcccagg tcgccattga tgcgggccag ctcgcggacg

901 tgctcatagt ccacgacgcc cgtgattttg tagccctggc cgacggccag caggtaggcc

961 gacaggctca tgccggccgc cgccgccttt tcctcaatcg ctcttcgttc gtctggaagg

1021 cagtacacct tgataggtgg gctgcccttc ctggttggct tggtttcatc agccatccgc

1081 ttgccctcat ctgttacgcc ggcggtagcc ggccagcctc gcagagcagg attcccgttg

1141 agcaccgcca ggtgcgaata agggacagtg aagaaggaac acccgctcgc gggtgggcct

1201 acttcaccta tcctgcccgg ctgacgccgt tggatacacc aaggaaagtc tacacgaacc

1261 ctttggcaaa atcctgtata tcgtgcgaaa aaggatggat ataccgaaaa aatcgctata

1321 atgaccccga agcagggtta tgcagcggaa gatgccattc gccattcagg ctgcgcaact

1381 gttgggaagg gcgatcggtg cgggcctctt cgctattacg ccagctggcg aaagggggat

1441 gtgctgcaag gcgattaagt tgggtaacgc cagggttttc ccagtcacga cgttgtaaaa

1501 cgacggccag tgaccatgat tacgccaagc tcgaaattaa ccctcactaa agggaacaaa

1561 agctggtacc taacaggatt agtgcaattc gagttgaatc actgggaaaa acattgtctt

1621 cttttttata ttatcatttg cattagtgct gcagtcgtag atacttgttg gttgaaagac

1681 atcagctggg agggactgga ctagcgtttg gtaaggagac atacctgtta acgttggttg

1741 caaaattcca tttcgcgatt tatgttatct gtaaatcctg atttgtctgg aattcttgat

1801 acttccgttt ttttagaggc caatgattag catcggcgat tctcaaaata gcattttcga

1861 catgcggtgc tgatttcata aacatagaca acgcttttac atgtaaaagt aacttgcgga

1921 cttggaacag tgctctgttt ttggtgtgaa cgtaactcag caatatttct gtgctagcaa

1981 ggttttttat gatcgaccga agatctcaaa actccgggtc tttcaactgt ctgactagac

2041 catgttcgta acgtcggaca gcagctttcg ttgtactggt agaatttcta cgtgcgaagc

2101 acgtgtaggc aggttgaacg acgatccctg ccgatggatg gattggcacg cggcggaacg

2161 ctttcgtgat ctacaccacc tggatcttca catatcttcg aaatcgaaaa attaaccaag

2221 tcgacggtat cgataatatt ctagctgagg gtacccatgg ccaagttgac cagtgccgtt

2281 ccggtgctca ccgcgcgcga cgtcgccgga gcggtcgagt tctggaccga ccggctcggg

2341 ttctcccggg acttcgtgga ggacgacttc gccggtgtgg tccgggacga cgtgaccctg

2401 ttcatcagcg cggtccagga ccaggtggtg ccggacaaca ccctggcctg ggtgtgggtg

2461 cgcggcctgg acgagctgta cgccgagtgg tcggaggtcg tgtccacgaa cttccgggac

2521 gcctccgggc cggccatgac cgagatcggc gagcagccgt gggggcggga gttcgccctg

2581 cgcgacccgg ccggcaactg cgtgcacttc gtggccgagg agcaggactg accgacgccg

2641 accaacaccg ccggtccgac gcggcccgac gggtccgagg cctcggagat ctgggcccat

2701 gcggccgcaa caactacctc gactttggct gggacacttt cagtgaggac aagaagcttc

2761 agaagcgtgc tatcgaactc aaccagggac gtgcggcaca aatgggcatc cttgctctca

2821 tggtgcacga acagttggga gtctctatcc ttccttaaaa atttaatttt cattagttgc

2881 agtcactccg ctttggtttc acagtcagga ataacactag ctcgtcttca atcacgtgct

2941 ataaaaataa ttataattta aattttttaa tataaatata taaattaaaa atagaaagta

3001 aaaaaagaaa ttaaagaaaa aatagttttt gttttccgaa gatgtaaaag actctagggg

3061 gatcgccaac aaatactacc ttttaccttg ctcttcctgc tctcaggtat taatgccgaa

3121 ttgtttcatc ttgtctgtgt agaagaccac acacgaaaat cctgtgattt tacattttac

3181 ttatcgttaa tcgaatgtat atctatttaa tctgcttttc ttgtctaata aatatatatg

3241 taaagtacgc tttttgttga aattttttaa acctttgttt attttttttt cttcattccg

3301 taactcttct accttcttta tttactttct aaaatccaaa tacaaaacat aaaaataaat

3361 aaacacagag taaattccca aattattcca tcattaaaag atacgaggcg cgtgtaagtt

3421 acaggcaagc gatcctagta cactctatat ttttttatgc ctcggtaatg attttcattt

3481 tttttttcca cctagcggat gactcttttt ttttcttagc gattggcatt atcacataat

3541 gaattataca ttatataaag taatgtgatt tcttcgaaga atatactaaa aaatgagcag

3601 gcaagataaa cgaaggcaaa gatgacagag cagactctag agtcgacctg cacatatgcc

3661 aaaatttcgt tcacggcagg gattttggcg tcgagatctt catgggacca aaactagcag

3721 gaggattggt ttttcagcat aatagattcg tgtccccgtt tcgatttttc tctgcggccg

3781 tccccaccgg acgctgaaaa atttcaaagc tgaagcattt ggtgaagtcg tacgccaagt

3841 ttcccaccag aaataatgtg gaccaagagg gttaagtgtc gcgcatgtgc gaagaatgcg

3901 ctgggagctg acttcccggc cgactgtaat tagagaagaa catagattaa aaaaagactt

3961 gtcaattcgg aagatacaca agtagacaca gaatactaaa caagaagaga gaagcatgac

4021 tcatcttttt aagcatcata atagatgatt tgcggggcgt ttgtatgagt cagccaagtg

4081 caaatatgtt ttacgtgtca acgaatctta aagatccgtg acaaaaacgc catacaccgt

4141 accatgaaat ggacgctttg ttaacgtaaa gtgtcgaatc ttcagtacac atttttcaag

4201 attcgctaga ttcgacagtg ataaatcatc tttgtcaaaa caatcttcat ggttgtcgaa

4261 cgatatacgg tctatacgtc ctaaatcaga gaaccgttca caaattacaa ttagattctt

4321 tagagaaacc atgaccaggc tcacgtgtac tttcttacaa ttgatgggat cggatgcaag

4381 accgaggaaa agatctcatc tcttgggcgc actgataaag ctcataattt tgtgagttgc

4441 caccaaggat ttttctatcg gcatcctaac attatcggac ctgcaaaact agtatggtga

4501 gcaagggcga ggagctgttc accggggtgg tgcccatcct ggtcgagctg gacggcgacg

4561 taaacggcca caagttcagc gtgtccggcg agggcgaggg cgatgccacc tacggcaagc

4621 tgaccctgaa gctgatctgc accaccggca agctgcccgt gccctggccc accctcgtga

4681 ccaccctggg ctacggcctg cagtgcttcg cccgctaccc cgaccacatg aagcagcacg

4741 acttcttcaa gtccgccatg cccgaaggct acgtccagga gcgcaccatc ttcttcaagg

4801 acgacggcaa ctacaagacc cgcgccgagg tgaagttcga gggcgacacc ctggtgaacc

4861 gcatcgagct gaagggcatc gacttcaagg aggacggcaa catcctgggg cacaagctgg

4921 agtacaacta caacagccac aacgtctata tcaccgccga caagcagaag aacggcatca

4981 aggccaactt caagatccgc cacaacatcg aggacggcgg cgtgcagctc gccgaccact

5041 accagcagaa cacccccatc ggcgacggcc ccgtgctgct gcccgacaac cactacctga

5101 gctaccagtc caagctgagc aaagacccca acgagaagcg cgatcacatg gtcctgctgg

5161 agttcgtgac cgccgccggg atcactctcg gcatggacga gctgtacaag taactacctc

5221 gactttggct gggacacttt cagtgaggac aagaagcttc agaagcgtgc tatcgaactc

5281 aaccagggac gtgcggcaca aatgggcatc cttgctctca tggtgcacga acagttggga

5341 gtctctatcc ttccttaaaa atttaatttt cattagttgc agtcactccg ctttggtttc

5401 acagtcagga ataacactag ctcgtcttca tgcaggcatc tcttccgctt cctcgctcac

5461 tgactcgctg cgctcggtcg ttcggctgcg gcgagcggta tcagctcact caaaggcggt

5521 aatacggtta tccacagaat caggggataa cgcaggaaag aacatgtgag caaaaggcca

5581 gcaaaaggcc aggaaccgta aaaaggccgc gttgctggcg tttttccata ggctccgccc

5641 ccctgacgag catcacaaaa atcgacgctc aagtcagagg tggcgaaacc cgacaggact

5701 ataaagatac caggcgtttc cccctggaag ctccctcgtg cgctctcctg ttccgaccct

5761 gccgcttacc ggatacctgt ccgcctttct cccttcggga agcgtggcgc tttctcatag

5821 ctcacgctgt aggtatctca gttcggtgta ggtcgttcgc tccaagctgg gctgtgtgca

5881 cgaacccccc gttcagcccg accgctgcgc cttatccggt aactatcgtc ttgagtccaa

5941 cccggtaaga cacgacttat cgccactggc agcagccact ggtaacagga ttagcagagc

6001 gaggtatgta ggcggtgcta cagagttctt gaagtggtgg cctaactacg gctacactag

6061 aaggacagta tttggtatct gcgctctgct gaagccagtt accttcggaa aaagagttgg

6121 tagctcttga tccggcaaac aaaccaccgc tggtagcggt ggtttttttg tttgcaagca

6181 gcagattacg cgcagaaaaa aaggatctca agaagatcct ttgatctttt ctacggggtc

6241 tgacgctcag tggaacgaaa actcacgtta agggattttg gtcatgagat tatcaaaaag

6301 gatcttcacc tagatccttt taaattaaaa atgaagtttt aaatcaatct aaagtatata

6361 tgagtaaact tggtctgaca gttaccaatg cttaatcagt gaggcaccta tctcagcgat

6421 ctgtctattt cgttcatcca tagttgcctg actccccgtc gtgtagataa ctacgatacg

6481 ggagggctta ccatctggcc ccagtgctgc aatgataccg cgagacccac gctcaccggc

6541 tccagattta tcagcaataa accagccagc cggaagggcc gagcgcagaa gtggtcctgc

6601 aactttatcc gcctccatcc agtctattaa ttgttgccgg gaagctagag taagtagttc

6661 gccagttaat agtttgcgca acgttgttgc cattgctgca ggcatcgtgg tgtcacgctc

6721 gtcgtttggt atggcttcat tcagctccgg ttcccaacga tcaaggcgag ttacatgatc

6781 ccccatgttg tgcaaaaaag cggttagctc cttcggtcct ccgatcgttg tcagaagtaa

6841 gttggccgca gtgttatcac tcatggttat ggcagcactg cataattctc ttactgtcat

6901 gccatccgta agatgctttt ctgtgactgg tgagtactca accaagtcat tctgagaata

6961 gtgtatgcgg cgaccgagtt gctcttgccc ggcgtcaaca cgggataata ccgcgccaca

7021 tagcagaact ttaaaagtgc tcatcattgg aaaacgttct tcggggcgaa aactctcaag

7081 gatcttaccg ctgttgagat ccagttcgat gtaacccact cgtgcaccca actgatcttc

7141 agcatctttt actttcacca gcgtttctgg gtgagcaaaa acaggaaggc aaaatgccgc

7201 aaaaaaggga ataagggcga cacggaaatg ttgaatactc atactcttcc tttttcaata

7261 ttattgaagc atttatcagg gttattgtct catgagcgga tacatatttg aatgtattta

7321 gaaaaataaa caaatagggg ttccgcgcac atttccccga aaagtgccac ctgacgtcta

7381 agaaaccatt attatcatga cattaaccta taaaaatagg cgtatcacga ggccctttcg

7441 tcttcaagaa ttctcatgtt tgacagctta tcatcgataa gctttaatgc ggtagtttat

7501 cacagttaaa ttgctaacgc agtcaggcac cgtgtatgaa atctaacaat gcgctcatcg

7561 tcatcctcgg caccgtcacc ctggatgctg taggcatagg cttggttatg ccggtactgc

7621 cgggcctctt gcgggatatc gtccattccg acagcatcgc cagtcactat ggcgtgctgc

7681 tagcgctata tgcgttgatg caatttctat gcgcacccgt tctcggagca ctgtccgacc

7741 gctttggccg ccgcccagtc ctgctcgctt cgctacttgg agccactatc gactacgcga

7801 tcatggcgac cacacccgtc ctgtggatcc tctacgccgg acgcatcgtg gccggcatca

7861 ccggcgccac aggtgcggtt gctggcgcct atatcgccga catcaccgat ggggaagatc

7921 gggctcgcca cttcgggctc atgagcgctt gtttcggcgt gggtatggtg gcaggccccg

7981 tggccggggg actgttgggc gccatctcct tgcatgcacc attccttgcg gcggcggtgc

8041 tcaacggcct caacctacta ctgggctgct tcctaatgca ggagtcgcat aagggagagc

8101 gtcgaccgat gcccttgaga gccttcaacc cagtcagctc cttccggtgg gcgcggggca

8161 tgactatcgt cgccgcactt atgactgtct tctttatcat gcaactcgta ggacaggtgc

8221 cggcagcgct ctgggtcatt ttcggcgagg accgctttcg ctggagcgcg acgatgatcg

8281 gcctgtcgct tgcggtattc ggaatcttgc acgccctcgc tcaagccttc gtcactggtc

8341 ccgccaccaa acgtttcggc gagaagcagg ccattatcgc cggcatggcg gccgacgcgc

8401 tgggctacgt cttgctggcg ttcgcgacgc gaggctggat ggccttcccc attatgattc

8461 ttctcgcttc cggcggcatc gggatgcccg cgttgcaggc catgctgtcc aggcaggtag

8521 atgacgacca tcagggacag cttcaaggat cgctcgcggc tcttaccagc ctaacttcga

8581 tcattggacc gctgatcgtc acggcgattt atgccgcctc ggcgagcaca tggaacgggt

8641 tggcatggat tgtaggcgcc gccctatacc ttgtctgcct ccccgcgttg cgtcgcggtg

8701 catggagccg ggccacctcg acctgaatgg aagccggcgg cacctcgcta acggattcac

8761 cactccaaga attggagcca atcaattctt gcggagaact gtgaatgcgc aaaccaaccc

8821 ttggcagaac atatccatcg cgtccgccat ctccagcagc cgcacgcggc gcatctcggg

8881 cagcgttggg tcctggccac gggtgcgcat gatcgtgctc ctgtcgttga ggacccggct

8941 aggctggcgg ggttgcctta ctggttagca gaatgaatca ccgatacgcg agcgaacgtg

9001 aagcgactgc tgctgcaaaa cgtctgcgac ctgagcaaca acatgaatgg tcttcggttt

9061 ccgtgtttcg taaagtctgg aa

//

LOCUS pUC19_AP1p_CrGES 5826 bp DNA circular 05-DEC-2019

DEFINITION .

ACCESSION urn.local...o-ba2hs82

KEYWORDS .

SOURCE

ORGANISM .

COMMENT Serial Cloner Genbank FormatCOMMENT SerialCloner_Type=DNA

COMMENT SerialCloner_Comments=Point

mutations in mVenus (2x) resault in same amino acid an d no protein

sequence change COMMENT

SerialCloner_Ends=0,0,,0,.

FEATURES Location/Qualifiers

promoter complement(767..795)

/note="Geneious type: Promoter"

/standard_name="Amp prom"

CDS complement(1164..1232)

/standard_name="LacZ alpha"

primer_bind 1303..1320

/standard_name="M13-fwd"

misc_feature 1363..2029

/standard_name="FCBPp"

CDS 2030..2404

/standard_name="Shble"

misc_feature 2405..2694

/standard_name="FCBPt"

misc_feature 2708..3535

/standard_name="pAP1p"

misc_feature 3553..4272

/standard_name="mVenus"

misc_feature 4273..4480

/standard_name="FCBPt"

primer_bind complement(4526..4546)

/standard_name="M13-rev"

misc_binding complement(4552..4574)

promoter complement(4579..4608)

/note="Geneious type: Promoter"

/standard_name="lac"

rep_origin complement(4914..5542)

ORIGIN

1 ccgcgagacc cacgctcacc ggctccagat ttatcagcaa taaaccagcc agccggaagg

61 gccgagcgca gaagtggtcc tgcaacttta tccgcctcca tccagtctat taattgttgc

121 cgggaagcta gagtaagtag ttcgccagtt aatagtttgc gcaacgttgt tgccattgct

181 acaggcatcg tggtgtcacg ctcgtcgttt ggtatggctt cattcagctc cggttcccaa

241 cgatcaaggc gagttacatg atcccccatg ttgtgcaaaa aagcggttag ctccttcggt

301 cctccgatcg ttgtcagaag taagttggcc gcagtgttat cactcatggt tatggcagca

361 ctgcataatt ctcttactgt catgccatcc gtaagatgct tttctgtgac tggtgagtac

421 tcaaccaagt cattctgaga atagtgtatg cggcgaccga gttgctcttg cccggcgtca

481 atacgggata ataccgcgcc acatagcaga actttaaaag tgctcatcat tggaaaacgt

541 tcttcggggc gaaaactctc aaggatctta ccgctgttga gatccagttc gatgtaaccc

601 actcgtgcac ccaactgatc ttcagcatct tttactttca ccagcgtttc tgggtgagca

661 aaaacaggaa ggcaaaatgc cgcaaaaaag ggaataaggg cgacacggaa atgttgaata

721 ctcatactct tcctttttca atattattga agcatttatc agggttattg tctcatgagc

781 ggatacatat ttgaatgtat ttagaaaaat aaacaaatag gggttccgcg cacatttccc

841 cgaaaagtgc cacctgacgt ctaagaaacc attattatca tgacattaac ctataaaaat

901 aggcgtatca cgaggccctt tcgtctcgcg cgtttcggtg atgacggtga aaacctctga

961 cacatgcagc tcccggagac ggtcacagct tgtctgtaag cggatgccgg gagcagacaa

1021 gcccgtcagg gcgcgtcagc gggtgttggc gggtgtcggg gctggcttaa ctatgcggca

1081 tcagagcaga ttgtactgag agtgcaccat atgcggtgtg aaataccgca cagatgcgta

1141 aggagaaaat accgcatcag gcgccattcg ccattcaggc tgcgcaactg ttgggaaggg

1201 cgatcggtgc gggcctcttc gctattacgc cagctggcga aagggggatg tgctgcaagg

1261 cgattaagtt gggtaacgcc agggttttcc cagtcacgac gttgtaaaac gacggccagt

1321 gaattcgagc tcggtacccg ggctaacagg attagtgcaa ttcgagttga atcactggga

1381 aaaacattgt cttctttttt atattatcat ttgcattagt gctgcagtcg tagatacttg

1441 ttggttgaaa gacatcagct gggagggact ggactagcgt ttggtaagga gacatacctg

1501 ttaacgttgg ttgcaaaatt ccatttcgcg atttatgtta tctgtaaatc ctgatttgtc

1561 tggaattctt gatacttccg tttttttaga ggccaatgat tagcatcggc gattctcaaa

1621 atagcatttt cgacatgcgg tgctgatttc ataaacatag acaacgcttt tacatgtaaa

1681 agtaacttgc ggacttggaa cagtgctctg tttttggtgt gaacgtaact cagcaatatt

1741 tctgtgctag caaggttttt tatgatcgac cgaagatctc aaaactccgg gtctttcaac

1801 tgtctgacta gaccatgttc gtaacgtcgg acagcagctt tcgttgtact ggtagaattt

1861 ctacgtgcga agcacgtgta ggcaggttga acgacgatcc ctgccgatgg atggattggc

1921 acgcggcgga acgctttcgt gatctacacc acctggatct tcacatatct tcgaaatcga

1981 aaaattaacc aagtcgacgg tatcgataat attctagctg agggtaccca tggccaagtt

2041 gaccagtgcc gttccggtgc tcaccgcgcg cgacgtcgcc ggagcggtcg agttctggac

2101 cgaccggctc gggttctccc gggacttcgt ggaggacgac ttcgccggtg tggtccggga

2161 cgacgtgacc ctgttcatca gcgcggtcca ggaccaggtg gtgccggaca acaccctggc

2221 ctgggtgtgg gtgcgcggcc tggacgagct gtacgccgag tggtcggagg tcgtgtccac

2281 gaacttccgg gacgcctccg ggccggccat gaccgagatc ggcgagcagc cgtgggggcg

2341 ggagttcgcc ctgcgcgacc cggccggcaa ctgcgtgcac ttcgtggccg aggagcagga

2401 ctgaccgacg ccgaccaaca ccgccggtcc gacgcggccc gacgggtccg aggcctcgga

2461 gatctgggcc catgcggccg caacaactac ctcgactttg gctgggacac tttcagtgag

2521 gacaagaagc ttcagaagcg tgctatcgaa ctcaaccagg gacgtgcggc acaaatgggc

2581 atccttgctc tcatggtgca cgaacagttg ggagtctcta tccttcctta aaaatttaat

2641 tttcattagt tgcagtcact ccgctttggt ttcacagtca ggaataacac tagctcgtct

2701 agtcgacctg cacatatgcc aaaatttcgt tcacggcagg gattttggcg tcgagattcg

2761 ttcatgggac caaacctagc aggaggattg gtttttcagc ataatagatt cgtgtccccg

2821 tttcgatttt tctcagcggc cgtccccact ggacgctgaa aaatttcaaa gttaaagcat

2881 ttggtgaaat cgtacgccaa gtttcccgct agaaataatg tggaccgagg gttaagtgtc

2941 gcgcatgtgc gaagaatgcg ctgggagctg acttcccggc cgactgtaat tagagaagaa

3001 catagattaa aaaaagactt gtcgattcgg aagacacaca agtagacaca gaatgctaaa

3061 caagaagaga gaagcatgac tcatcttttt aagcatcata atagatgatt tgcggggcgt

3121 ttgtatgagt cagccaagtg caaatatgtt ttacgtgtca acgaatctta agatccgtga

3181 caaaaacccc atacaccgta ccatgaaatg gacgctttgt taacgtaaag tgtcgaatct

3241 tcagtacaca tttttcaaga ttcgctagat tcgacagtga taaatcatct ttgtcaaaac

3301 aatcttcatg gttgtcgaac gatatacggt ctatacgtcc taaatcagag aaccgttcac

3361 aaattacaat tagattcttt agagaaacca tgaccaggct cacgtgtact ttcttacaat

3421 tgatgggatc ggatgcaaga ccgaggaaaa gatctcatct cttgggcgca ctgataaagc

3481 tcataatttt gtgagttgcc accaaggatt tttctatcgg catcctaaca ttatcggacc

3541 tgcaaaacta gtatggtgag caagggcgag gagctgttca ccggggtggt gcccatcctg

3601 gtcgagctgg acggcgacgt aaacggacac aagttcagcg tgtccggcga gggcgagggc

3661 gatgccacct acggcaagct gaccctgaag ctgatctgca ccaccggcaa gctgcccgtg

3721 ccctggccca ccctcgtgac caccctggga tacggcctgc agtgcttcgc ccgctacccc

3781 gaccacatga agcagcacga cttcttcaag tccgccatgc ccgaaggcta cgtccaggag

3841 cgcaccatct tcttcaagga cgacggcaac tacaagaccc gcgccgaggt gaagttcgag

3901 ggcgacaccc tggtgaaccg catcgagctg aagggcatcg acttcaagga ggacggcaac

3961 atcctggggc acaagctgga gtacaactac aacagccaca acgtctatat caccgccgac

4021 aagcagaaga acggcatcaa ggccaacttc aagatccgcc acaacatcga ggacggcggc

4081 gtgcagctcg ccgaccacta ccagcagaac acccccatcg gcgacggccc cgtgctgctg

4141 cccgacaacc actacctgag ctaccagtcc aagctgagca aagaccccaa cgagaagcgc

4201 gatcacatgg tcctgctgga gttcgtgacc gccgccggga tcactctcgg catggacgag

4261 ctgtacaagt aactacctcg actttggctg ggacactttc agtgaggaca agaagcttca

4321 gaagcgtgct atcgaactca accagggacg tgcggcacaa atgggcatcc ttgctctcat

4381 ggtgcacgaa cagttgggag tctctatcct tccttaaaaa tttaattttc attagttgca

4441 gtcactccgc tttggtttca cagtcaggaa taacactagc tcgtctctct agagtcgacc

4501 tgcaggcatg caagcttggc gtaatcatgg tcatagctgt ttcctgtgtg aaattgttat

4561 ccgctcacaa ttccacacaa catacgagcc ggaagcataa agtgtaaagc ctggggtgcc

4621 taatgagtga gctaactcac attaattgcg ttgcgctcac tgcccgcttt ccagtcggga

4681 aacctgtcgt gccagctgca ttaatgaatc ggccaacgcg cggggagagg cggtttgcgt

4741 attgggcgct cttccgcttc ctcgctcact gactcgctgc gctcggtcgt tcggctgcgg

4801 cgagcggtat cagctcactc aaaggcggta atacggttat ccacagaatc aggggataac

4861 gcaggaaaga acatgtgagc aaaaggccag caaaaggcca ggaaccgtaa aaaggccgcg

4921 ttgctggcgt ttttccatag gctccgcccc cctgacgagc atcacaaaaa tcgacgctca

4981 agtcagaggt ggcgaaaccc gacaggacta taaagatacc aggcgtttcc ccctggaagc

5041 tccctcgtgc gctctcctgt tccgaccctg ccgcttaccg gatacctgtc cgcctttctc

5101 ccttcgggaa gcgtggcgct ttctcatagc tcacgctgta ggtatctcag ttcggtgtag

5161 gtcgttcgct ccaagctggg ctgtgtgcac gaaccccccg ttcagcccga ccgctgcgcc

5221 ttatccggta actatcgtct tgagtccaac ccggtaagac acgacttatc gccactggca

5281 gcagccactg gtaacaggat tagcagagcg aggtatgtag gcggtgctac agagttcttg

5341 aagtggtggc ctaactacgg ctacactaga agaacagtat ttggtatctg cgctctgctg

5401 aagccagtta ccttcggaaa aagagttggt agctcttgat ccggcaaaca aaccaccgct

5461 ggtagcggtg gtttttttgt ttgcaagcag cagattacgc gcagaaaaaa aggatctcaa

5521 gaagatcctt tgatcttttc tacggggtct gacgctcagt ggaacgaaaa ctcacgttaa

5581 gggattttgg tcatgagatt atcaaaaagg atcttcacct agatcctttt aaattaaaaa

5641 tgaagtttta aatcaatcta aagtatatat gagtaaactt ggtctgacag ttaccaatgc

5701 ttaatcagtg aggcacctat ctcagcgatc tgtctatttc gttcatccat agttgcctga

5761 ctccccgtcg tgtagataac tacgatacgg gagggcttac catctggccc cagtgctgca

5821 atgata

//

LOCUS pUC19_AP1p_CrGES 7593 bp DNA circular 05-DEC-2019

DEFINITION .

ACCESSION urn.local...k-ba2hqlz

KEYWORDS .

SOURCE

ORGANISM .

COMMENT Serial Cloner Genbank FormatCOMMENT SerialCloner_Type=DNA

COMMENT SerialCloner_Comments=

COMMENT SerialCloner_Ends=0,0,,0,.

FEATURES Location/Qualifiers

CDS complement(1..527)

/standard_name="AmpR [*]"

promoter complement(343..371)

/note="Geneious type: Promoter"

/standard_name="Amp prom"

CDS complement(740..808)

/standard_name="LacZ alpha"

primer_bind 1303..1320

/standard_name="M13-fwd"

misc_feature 1363..2029

/standard_name="FCBPp"

CDS 2030..2404

/standard_name="Shble"

misc_feature 2405..2694

/standard_name="FCBPt"

misc_feature 2708..3535

/standard_name="pAP1p"

misc_feature 3553..5319

/standard_name="GeS"

misc_feature 5320..6039

/standard_name="mVenus"

misc_feature 6041..6247

/standard_name="FCBPt"

primer_bind complement(6293..6313)

/standard_name="M13-rev"

misc_binding complement(6319..6341)

promoter complement(6346..6375)

/note="Geneious type: Promoter"

/standard_name="lac"

rep_origin complement(6681..6694)

ORIGIN

1 ccgcgagacc cacgctcacc ggctccagat ttatcagcaa taaaccagcc agccggaagg

61 gccgagcgca gaagtggtcc tgcaacttta tccgcctcca tccagtctat taattgttgc

121 cgggaagcta gagtaagtag ttcgccagtt aatagtttgc gcaacgttgt tgccattgct

181 acaggcatcg tggtgtcacg ctcgtcgttt ggtatggctt cattcagctc cggttcccaa

241 cgatcaaggc gagttacatg atcccccatg ttgtgcaaaa aagcggttag ctccttcggt

301 cctccgatcg ttgtcagaag taagttggcc gcagtgttat cactcatggt tatggcagca

361 ctgcataatt ctcttactgt catgccatcc gtaagatgct tttctgtgac tggtgagtac

421 tcaaccaagt cattctgaga atagtgtatg cggcgaccga gttgctcttg cccggcgtca

481 atacgggata ataccgcgcc acatagcaga actttaaaag tgctcatcat tggaaaacgt

541 tcttcggggc gaaaactctc aaggatctta ccgctgttga gatccagttc gatgtaaccc

601 actcgtgcac ccaactgatc ttcagcatct tttactttca ccagcgtttc tgggtgagca

661 aaaacaggaa ggcaaaatgc cgcaaaaaag ggaataaggg cgacacggaa atgttgaata

721 ctcatactct tcctttttca atattattga agcatttatc agggttattg tctcatgagc

781 ggatacatat ttgaatgtat ttagaaaaat aaacaaatag gggttccgcg cacatttccc

841 cgaaaagtgc cacctgacgt ctaagaaacc attattatca tgacattaac ctataaaaat

901 aggcgtatca cgaggccctt tcgtctcgcg cgtttcggtg atgacggtga aaacctctga

961 cacatgcagc tcccggagac ggtcacagct tgtctgtaag cggatgccgg gagcagacaa

1021 gcccgtcagg gcgcgtcagc gggtgttggc gggtgtcggg gctggcttaa ctatgcggca

1081 tcagagcaga ttgtactgag agtgcaccat atgcggtgtg aaataccgca cagatgcgta

1141 aggagaaaat accgcatcag gcgccattcg ccattcaggc tgcgcaactg ttgggaaggg

1201 cgatcggtgc gggcctcttc gctattacgc cagctggcga aagggggatg tgctgcaagg

1261 cgattaagtt gggtaacgcc agggttttcc cagtcacgac gttgtaaaac gacggccagt

1321 gaattcgagc tcggtacccg ggctaacagg attagtgcaa ttcgagttga atcactggga

1381 aaaacattgt cttctttttt atattatcat ttgcattagt gctgcagtcg tagatacttg

1441 ttggttgaaa gacatcagct gggagggact ggactagcgt ttggtaagga gacatacctg

1501 ttaacgttgg ttgcaaaatt ccatttcgcg atttatgtta tctgtaaatc ctgatttgtc

1561 tggaattctt gatacttccg tttttttaga ggccaatgat tagcatcggc gattctcaaa

1621 atagcatttt cgacatgcgg tgctgatttc ataaacatag acaacgcttt tacatgtaaa

1681 agtaacttgc ggacttggaa cagtgctctg tttttggtgt gaacgtaact cagcaatatt

1741 tctgtgctag caaggttttt tatgatcgac cgaagatctc aaaactccgg gtctttcaac

1801 tgtctgacta gaccatgttc gtaacgtcgg acagcagctt tcgttgtact ggtagaattt

1861 ctacgtgcga agcacgtgta ggcaggttga acgacgatcc ctgccgatgg atggattggc

1921 acgcggcgga acgctttcgt gatctacacc acctggatct tcacatatct tcgaaatcga

1981 aaaattaacc aagtcgacgg tatcgataat attctagctg agggtaccca tggccaagtt

2041 gaccagtgcc gttccggtgc tcaccgcgcg cgacgtcgcc ggagcggtcg agttctggac

2101 cgaccggctc gggttctccc gggacttcgt ggaggacgac ttcgccggtg tggtccggga

2161 cgacgtgacc ctgttcatca gcgcggtcca ggaccaggtg gtgccggaca acaccctggc

2221 ctgggtgtgg gtgcgcggcc tggacgagct gtacgccgag tggtcggagg tcgtgtccac

2281 gaacttccgg gacgcctccg ggccggccat gaccgagatc ggcgagcagc cgtgggggcg

2341 ggagttcgcc ctgcgcgacc cggccggcaa ctgcgtgcac ttcgtggccg aggagcagga

2401 ctgaccgacg ccgaccaaca ccgccggtcc gacgcggccc gacgggtccg aggcctcgga

2461 gatctgggcc catgcggccg caacaactac ctcgactttg gctgggacac tttcagtgag

2521 gacaagaagc ttcagaagcg tgctatcgaa ctcaaccagg gacgtgcggc acaaatgggc

2581 atccttgctc tcatggtgca cgaacagttg ggagtctcta tccttcctta aaaatttaat

2641 tttcattagt tgcagtcact ccgctttggt ttcacagtca ggaataacac tagctcgtct

2701 agtcgacctg cacatatgcc aaaatttcgt tcacggcagg gattttggcg tcgagattca

2761 tgggaccaaa actagcagga ggattggttt ttcagcataa tagattcgtg tccccgtttc

2821 gatttttctc tgcggccgtc cccaccggac gctgaaaaat ttcaaagctg aagcatttgg

2881 tgaagtcgta cgccaagttt cccaccagaa ataatgtgga ccaagaaggg ttaagtgtcg

2941 cgcatgtgcg aagaatgcgc tgggagctga cttcccggcc gactgtaatt agagaagaac

3001 atagattaaa aaagacttgt caattcggaa gatacacaag tagacacaga atactaaaca

3061 agaagagaga agcatgactc atctttttaa gcatcataat agatgatttg cagggcgttt

3121 gtatgagtca gccaagtgca aatatgtttt acgtgtcaac gaatcttaaa gatccatgac

3181 aaaaacgcca tacaccagaa ccatgaaatg gacgcttcgt taatgtaaag tgtcgaatct

3241 tcagtacaca tttttcaaga ttcgctagat tcgacagtga taaatcatct ttgtcaaaac

3301 aatcttcatg gttgttgaac gatatacggt ctatacgtcc taaatcagag aaccgttcac

3361 aaattacaat tagattcttt agagaaacca tgaccaggct cacgtgtact ttcttacaat

3421 tgatgggatc ggatgcaaga ccgaggaaaa gatctcatct cttgggcgca ccgataaagc

3481 tcataatttt gtgagttgcc accaaggatt tttctatcgg catcctaaca ttatcggacc

3541 tgcaaaacta gtatggccgc tacgatcagt aacttgtcgt ttttggctaa gtcacgcgca

3601 ttgtctcgcc cttcttctag ttcgctttct tggcttgaac gcccgaaaac ttcgtcaact

3661 atctgtatgt ccatgccctc aagttcctcg tctagttcat cttcctctat gtcccttcca

3721 cttgcaacac ctctcattaa agacaacgaa tcacttatta agtttctcag acaacccttg

3781 gttctccctc atgaggtaga cgactcgaca aaacgtcgag agttgttgga gagaacccgc

3841 aaagaattgg agcttaacgc cgaaaaaccg ttggaggcac tcaaaatgat cgacatcatc

3901 cagagactcg gactttcgta tcactttgag gatgatatca attcaatcct tacaggcttt

3961 agtaacattt cgagtcagac gcacgaagac ctcttgactg ctagcctctg cttcagactc

4021 ttgcgacaca acggccataa gattaatcca gatattttcc aaaaatttat ggacaataac

4081 ggaaagttca aagattcgct taaggacgac acattgggta tgctctcgct ctacgaagct

4141 tcgtatctcg gagcaaacgg cgaggagatc cttatggagg ctcaagagtt tacaaagacc

4201 caccttaaaa attcgttgcc agcaatggct ccatctcttt caaaaaaggt tagccaagca

4261 cttgaacagc cccgccaccg tcgtatgctc cgcctcgagg caaggcgatt tatcgaagaa

4321 tatggggcgg agaacgacca taatcccgac ttgcttgaat tggccaagct tgattacaat

4381 aaggttcaaa gcttgcacca gatggagctt agtgagatca ctcgctggtg gaaacagctc

4441 gggttggtcg acaaacttac gttcgccagg gatcgaccgc ttgaatgctt tctctggacc

4501 gtagggttgc ttccagagcc caagtatagt ggttgcagaa tcgaacttgc aaaaaccatt

4561 gccattcttc ttgtcattga cgatattttc gatacgcacg gcacccttga tgaattgttg

4621 ctctttacga atgcgatcaa gagatgggac cttgaagcga tggaagatct cccagagtat

4681 atgaggatct gctatatggc cctttacaat acgacaaacg aaatttgcta taaagtattg

4741 aaggaaaacg gttggtcggt ccttccgtat cttaaagcga cgtggattga catgatcgag

4801 gggtttatgg ttgaagctga atggtttaac tccgattacg tccccaatat ggaagaatac

4861 gtggagaacg gagtgcgaac cgcaggatct tacatggcgc ttgtccactt gtttttcctc

4921 attggccaag gcgtcactga ggataacgtg aaacttctca ttaagcccta tccaaagctt

4981 tttagcagct caggacgtat tttgcgactc tgggatgatt tgggtacggc taaggaagaa

5041 caggaacgcg gagatctcgc ttcctcaatc caacttttta tgagggagaa agagattaag

5101 tcagaagagg agggcagaaa ggggatcttg gagatcattg agaacctctg gaaagagctt

5161 aacggtgaac ttgtataccg cgaggagatg ccgcttgcga tcatcaaaac ggcattcaat

5221 atggcccgtg cttcacaggt ggtttatcaa cacgaagagg acacttattt tagttctgtt

5281 gacaactatg taaaagcatt gtttttcacg ccctgcttca tggtgagcaa gggcgaggag

5341 ctgttcaccg gggtggtgcc catcctggtc gagctggacg gcgacgtaaa cggacacaag

5401 ttcagcgtgt ccggcgaggg cgagggcgat gccacctacg gcaagctgac cctgaagctg

5461 atctgcacca ccggcaagct gcccgtgccc tggcccaccc tcgtgaccac cctgggatac

5521 ggcctgcagt gcttcgcccg ctaccccgac cacatgaagc agcacgactt cttcaagtcc

5581 gccatgcccg aaggctacgt ccaggagcgc accatcttct tcaaggacga cggcaactac

5641 aagacccgcg ccgaggtgaa gttcgagggc gacaccctgg tgaaccgcat cgagctgaag

5701 ggcatcgact tcaaggagga cggcaacatc ctggggcaca agctggagta caactacaac

5761 agccacaacg tctatatcac cgccgacaag cagaagaacg gcatcaaggc caacttcaag

5821 atccgccaca acatcgagga cggcggcgtg cagctcgccg accactacca gcagaacacc

5881 cccatcggcg acggccccgt gctgctgccc gacaaccact acctgagcta ccagtccaag

5941 ctgagcaaag accccaacga gaagcgcgat cacatggtcc tgctggagtt cgtgaccgcc

6001 gccgggatca ctctcggcat ggacgagctg tacaagtaac tacctcgact ttggctggga

6061 cactttcagt gaggacaaga agcttcagaa gcgtgctatc gaactcaacc agggacgtgc

6121 ggcacaaatg ggcatccttg ctctcatggt gcacgaacag ttgggagtct ctatccttcc

6181 ttaaaaattt aattttcatt agttgcagtc actccgcttt ggtttcacag tcaggaataa

6241 cactagctcg tctctctaga gtcgacctgc aggcatgcaa gcttggcgta atcatggtca

6301 tagctgtttc ctgtgtgaaa ttgttatccg ctcacaattc cacacaacat acgagccgga

6361 agcataaagt gtaaagcctg gggtgcctaa tgagtgagct aactcacatt aattgcgttg

6421 cgctcactgc ccgctttcca gtcgggaaac ctgtcgtgcc agctgcatta atgaatcggc

6481 caacgcgcgg ggagaggcgg tttgcgtatt gggcgctctt ccgcttcctc gctcactgac

6541 tcgctgcgct cggtcgttcg gctgcggcga gcggtatcag ctcactcaaa ggcggtaata

6601 cggttatcca cagaatcagg ggataacgca ggaaagaaca tgtgagcaaa aggccagcaa

6661 aaggccagga accgtaaaaa ggccgcgttg ctggcgtttt tccataggct ccgcccccct

6721 gacgagcatc acaaaaatcg acgctcaagt cagaggtggc gaaacccgac aggactataa

6781 agataccagg cgtttccccc tggaagctcc ctcgtgcgct ctcctgttcc gaccctgccg

6841 cttaccggat acctgtccgc ctttctccct tcgggaagcg tggcgctttc tcatagctca

6901 cgctgtaggt atctcagttc ggtgtaggtc gttcgctcca agctgggctg tgtgcacgaa

6961 ccccccgttc agcccgaccg ctgcgcctta tccggtaact atcgtcttga gtccaacccg

7021 gtaagacacg acttatcgcc actggcagca gccactggta acaggattag cagagcgagg

7081 tatgtaggcg gtgctacaga gttcttgaag tggtggccta actacggcta cactagaaga

7141 acagtatttg gtatctgcgc tctgctgaag ccagttacct tcggaaaaag agttggtagc

7201 tcttgatccg gcaaacaaac caccgctggt agcggtggtt tttttgtttg caagcagcag

7261 attacgcgca gaaaaaaagg atctcaagaa gatcctttga tcttttctac ggggtctgac

7321 gctcagtgga acgaaaactc acgttaaggg attttggtca tgagattatc aaaaaggatc

7381 ttcacctaga tccttttaaa ttaaaaatga agttttaaat caatctaaag tatatatgag

7441 taaacttggt ctgacagtta ccaatgctta atcagtgagg cacctatctc agcgatctgt

7501 ctatttcgtt catccatagt tgcctgactc cccgtcgtgt agataactac gatacgggag

7561 ggcttaccat ctggccccag tgctgcaatg ata

//
